# Gene Expression Changes in the Motor Cortex Mediating Motor Skill Learning

**DOI:** 10.1371/journal.pone.0061496

**Published:** 2013-04-24

**Authors:** Vincent C. K. Cheung, Caroline DeBoer, Elizabeth Hanson, Marta Tunesi, Mara D'Onofrio, Ivan Arisi, Rossella Brandi, Antonino Cattaneo, Ki A. Goosens

**Affiliations:** 1 McGovern Institute for Brain Research, and Department of Brain and Cognitive Sciences, Massachusetts Institute of Technology, Cambridge, Massachusetts, United States of America; 2 Department of Chemistry, Materials and Chemical Engineering ‘Giulio Natta’, Politecnico di Milano, Milan, Italy; 3 European Brain Research Institute ‘Rita Levi-Montalcini’, Rome, Italy; University of Nebraska Medical Center, United States of America

## Abstract

The primary motor cortex (M1) supports motor skill learning, yet little is known about the genes that contribute to motor cortical plasticity. Such knowledge could identify candidate molecules whose targeting might enable a new understanding of motor cortical functions, and provide new drug targets for the treatment of diseases which impair motor function, such as ischemic stroke. Here, we assess changes in the motor-cortical transcriptome across different stages of motor skill acquisition. Adult rats were trained on a gradually acquired appetitive reach and grasp task that required different strategies for successful pellet retrieval, or a sham version of the task in which the rats received pellet reward without needing to develop the reach and grasp skill. Tissue was harvested from the forelimb motor-cortical area either before training commenced, prior to the initial rise in task performance, or at peak performance. Differential classes of gene expression were observed at the time point immediately preceding motor task improvement. Functional clustering revealed that gene expression changes were related to the synapse, development, intracellular signaling, and the fibroblast growth factor (FGF) family, with many modulated genes known to regulate synaptic plasticity, synaptogenesis, and cytoskeletal dynamics. The modulated expression of synaptic genes likely reflects ongoing network reorganization from commencement of training till the point of task improvement, suggesting that motor performance improves only after sufficient modifications in the cortical circuitry have accumulated. The regulated FGF-related genes may together contribute to M1 remodeling through their roles in synaptic growth and maturation.

## Introduction

The mammalian brain is endowed with a tremendously flexible motor system that enables the individual to learn new motor skills throughout its adult span. The primary motor cortex (M1) is the brain region believed to support the acquisition and retention of motor memory by storing task-specific representations of new motor skills [1−5]. Previous experiments have suggested that M1 neuronal circuits undergo functional remodeling in response to skill training. Rats trained on a forelimb task exhibited a reorganized motor-cortical map with an expanded wrist-and-finger representation [6]; experiments using M1 slices have demonstrated training-induced strengthening of synaptic connections through a mechanism similar to long-term potentiation (LTP) [7−8]; imaging studies have also shown that after skill training, new M1 synapses are formed and stabilized [9−10], indicative of neuronal rewiring. As a result of M1's extensive connections with brainstem and spinal interneurons [11] and of the substantial intermingling of the cortico-motoneurons for different muscles within M1 [12], plastic rearrangement of the M1 circuitry may allow the emergence of new motor patterns, through differential recruitment of either existing or newly-formed muscle synergies, for executing the learned motor behavior.

Reorganization of cortical circuits likely requires transcriptional changes in many genes, including those involved in neurite outgrowth, modification of dendritic morphology, and synapse stabilization. Experience-dependent gene transcription responses have been demonstrated in multiple cortical regions following spatial learning [13], and in the primary visual cortex during the critical period [14]. It is thus possible that in M1, during motor skill learning, there exists training-dependent transcriptional regulation for an ensemble of genes that ultimately enables the improvement and consolidation of task performance between practice sessions.

Here, we ask the question of whether there is slow, accumulative change in motor cortical gene expression that underlies inter-session performance gain at different time points of motor skill learning. We first designed a behavioral task in which adult rats were trained to reach and grasp objects presented at randomized locations using their preferred forelimb. Across-day improvement in task performance followed a sigmoid time course, which permitted us to sample the transcriptome of the forelimb area of the motor cortex at three distinct time points on the learning curve: before training commenced, immediately before task performance improved, and after performance reached a plateau. Functional analysis of the gene expression profiles obtained using whole-genome microarrays identified many differentially expressed genes related to the synapse and growth-factor families that may contribute to circuitry reorganization. A qualitative correlation between the time course of motor behavior and the expression dynamics of the synaptic genes further allowed us to gain insights into the temporal relationship between neuronal remodeling in M1 and behavioral changes.

## Materials and Methods

### Ethics Statement

All experimental procedures were reviewed and approved by the MIT Committee on Animal Care (Protocol Number: 0910-073-13), and were designed in accordance with the recommendations in the Guide for the Care and Use of Laboratory Animals of the National Institutes of Health, USA. Full efforts were made to minimize suffering and discomfort of the animals.

### Rat Behavioral Tasks

**Figure 1:**
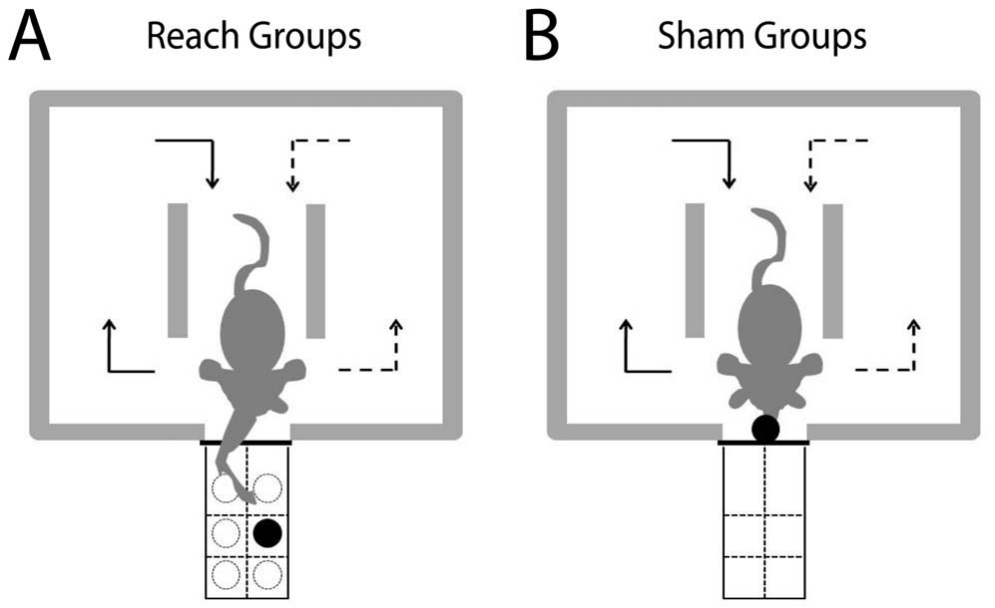
A nontrivial forelimb reach-and-grasp task for the rat. Adult rats were trained to perform either a forelimb reach-and-grasp task (*Reach* groups) or a sham version of the task (*Sham* groups) for either 5 days (*Reach*, N = 4; *Sham*, N = 4) or 12 days (*Reach*, N = 4; *Sham*, N = 4) before tissue harvest. *A*, Behavioral setup for the *Reach* groups. In each trial a food pellet was placed at one of six possible slots arranged in three rows at different distances from the animal (near, mid, and far slots) and two columns that were ipsilateral and contralateral to the animal's preferred limb, respectively. The task goal was to reach and grasp the pellet using the preferred limb with single or multiple reaches. A reach was defined to be any hand trajectory from the box to the shelf area that crossed the border between the box and shelf (indicated by the thick black horizontal line), and then back to the box by crossing the same line. *B*, Behavioral setup for the *Sham* groups. In each trial the pellet was placed in the slit. Because the pellet was easily accessible, the rat naturally retrieved the reward with its tongue without using its forelimb. In all groups, the animal was conditioned to initiate a new trial by turning down either the left (left-handed rats, dotted-line arrows) or right (right-handed rats, solid-line arrows) aisle from the central aisle.

Adult male Long-Evans rats (∼300 g, 2–3 months old) were trained to reach and grasp for a food pellet (20 mg; BioServ, Frenchtown, NJ) placed at a variable location using the preferred limb (*Reach* group). Training occurred in a Plexiglas box (width of 30 cm; length of 45 cm; height of 30 cm). A vertical slit opening (width of 1 cm) on the box's front allowed the rat to extend its forelimb to reach for the pellet placed on a shelf (height of 1.8 cm) in front of the slit. In each trial, the pellet was randomly assigned to one of six possible slots, arranged in three rows whose centers were 1.4, 1.9, and 2.4 cm from the inner side of the box's front wall, respectively. The outer left and right borders of each row aligned with the left and right borders of the slit opening (Fig. 1A). The slots were demarcated by markings on the shelf without any indentation on the shelf floor. By randomly varying the pellet location in each trial and placing the pellet on a flat surface without any indentation so that the pellet was more prone to being knocked away unless it was firmly grasped, we made the task not only more difficult for the rat to master, but also a better model of the learning of a novel skill that requires flexible adjustment of motor output. Our paradigm thus differs from the standard rodent forelimb task which demands the animal to reach and grasp from a single, fixed location in the workspace (e.g., [10], [15]).

The interior of the behavioral box was divided into three aisles. Two walls separated the central from the left and right aisles, respectively, and the slit opening was centered at the front of the center aisle. To prevent the rat from starting its reach before the pellet was properly placed on the shelf, after each trial the rat was trained to turn down the left aisle for left-handed rats, or the right aisle for right-handed rats (see below for a description of how handedness of each rat was determined), and then, re-approach the slit through the central aisle to initiate the next trial.

A total of 11 rats were trained on this forelimb task for 12 days, but motor cortical tissues were harvested from 4 of them (*12-day-Reach* group; see below) for microarray analysis. An additional group of animals serving as controls (*Sham* group) was trained to perform a version of the above task in which the food pellet was placed not on the shelf, but within the slit opening (Fig. 1B). Because the pellet was easily accessible, rats in the *Sham* group retrieved the reward with the tongue, without the use of the forelimb. Like the forelimb-trained rats in the *Reach* group, the *Sham* rats were also trained to exit the pellet area via the side aisle corresponding to the handedness of the individual rat. Thus the experimental and sham versions of the task were identical, except that rats in the *Reach* group had to acquire forelimb motor skills for successful pellet retrieval.

### Experimental Design

Throughout the experiment, the rats were maintained on a 12/12 h light-dark cycle, and fed daily with rat chow (12–18 g) after their behavioral session. Before commencement of forelimb training, we determined the rat's handedness by counting the frequency of use of each limb as the rat attempted to retrieve pellets on the shelf. For this determination we put multiple pellets at non-discrete positions very close to the slit opening, so that their retrieval would not require any learned motor skill. The rat was considered to prefer one of the two limbs if, for two consecutive days with at least 50 reaches displayed in each day, more than 80% of the reaches were from one side. All animals used in this study showed handedness (left-handed, 12 rats; right-handed, 15 rats). After confirmation of handedness, the animal was trained, through operant conditioning, to turn down the appropriate side aisle to initiate each experimental trial. During this training, whenever the rat displayed the correct behavior, it was positively reinforced with pellets placed inside the slit, which were invariably licked instead of grasped. This ensured that no animal was prematurely exposed to the forelimb task during this training of a task contingency. The rat was considered to have learned how to initiate trials when, within a single session, it displayed 20 consecutive turns for a 1-pellet reward for each turn.

Twenty rats divided into five groups of four were studied and sacrificed at different time points. Rats in the *0-day* group were sacrificed the day after they had learned trial initiation and were never exposed to the reach-and-grasp portion of the task. Rats in the *5-day-Reach* and *12-day-Reach* groups were trained with the forelimb task for 5 and 12 consecutive days, respectively, before motor cortical tissue was harvested. Similarly, the *5-day-Sham* and *12-day-Sham* groups were trained with the sham version of the task.

For all groups except *0-day,* the rat was trained with 120 trials every day, consisting of 20 daily trials per slot for the *5-* and *12-day-Reach* groups. The ordering of the slots in each day followed a randomized sequence generated before the behavioral session by the randperm function of Matlab (Mathworks, Natick, MA) such that every slot was uniformly distributed across the trials of the day. Since the reward for each trial consisted only of a single 20-mg pellet, the maximum amount of additional food a rat could earn during a session was 120×0.02 g = 2.4 g, a small amount compared with the rats' daily ration of regular chow (12–18 g). Thus, the difference in overall food intake between the *Reach-* and *Sham-*rats was expected to be small, if present at all. All sessions were videotaped (Sony DCR-HC46) for offline behavioral analysis. The start and end times of every session were recorded for estimation of the average trial duration. The weight of each rat was also measured at least once per week.

### Tissue Harvest

Each animal was sacrificed for the harvest of its forelimb motor cortex approximately 24 h after its last behavioral session, so that gene expression would reflect stable, cumulative changes of the transcriptome rather than transient changes that occur as the result of an individual training session. By harvesting only the forelimb motor cortex, the transcriptomic changes would also primarily reflect genes associated with the fine motor aspects of the reach-and-grasp task, rather than the motor skills required for locomotion through the apparatus, or consumption of the pellet. The animal was anesthetized with isoflurane before its brain was rapidly removed from the cranium. The brain was then placed, dorsal side up, on an ice-chilled brain matrix. Coronal sections were generated by placing razor blades at 0 and +4 mm anterior to Bregma, which correspond to the posterior and anterior boundaries of the rat forelimb motor areas, respectively [16]. This dissected brain section was immediately immersed into 5 mL of RNAlater (Qiagen, Valencia, CA) for RNA stabilization and stored at −20°C. The forelimb motor areas contralateral to the rat's preferred side was subsequently dissected out from this RNAlater-stabilized section under a microscope with parasagittal cuts at 2 and 5mm lateral to Bregma, and a horizontal cut at the gray-white matter border.

Importantly, in addition to the 20 animals, the frontal brain section of an age-matched, naïve rat not having been exposed to either the experimental or sham version of the task was similarly harvested and stabilized. In subsequent 2-color microarray hybridizations, the RNA extracted from this tissue provided a common reference channel for all 20 samples.

### RNA Extraction and Microarray Processing

In our microarray experiment, the motor cortical transcriptome of each animal (*Reach* or *Sham*) was hybridized to a single 2-color gene array; thus, a total of 20 microarrays (Agilent whole rat genome; G4131F; design ID: 01479; 41,012 unique biological probes) were employed. Each array consisted of two channels: cyanine-5 (cy5) and cyanine-3 (cy3). The cy5 channel of each array was linked to a sample from one of the 20 rats. The cy3 channel of all arrays was linked to the same sample from the frontal section of a naïve rat so that all microarrays were grounded to a common reference.

Total RNA was extracted from the dissected forelimb motor cortex using the Qiagen miRNeasy Mini kit, and purified with on-column digestion of DNA using RNase-free DNase (Qiagen). The quantity and integrity of the extracted RNA was assessed using the Agilent 2100 Bioanalyzer, which confirmed that all RNA samples were of high quality (RNA Integrity Number≥8.5). Gene expression profiling was then accomplished by 2-color hybridizations to the Agilent arrays. Using both T7 RNA polymerase and double-stranded cDNA synthesized from the sample RNA, cRNA with incorporated cyanine-labeled CTP was synthesized. Each of the cy5-labeled cRNA sample and the cy3-labeled reference cRNA were then mixed and hybridized to an oligonucleotide microarray per Agilent specifications. Hybridized arrays were then scanned using Agilent's G2505B Microarray Scanner System, and expression data were extracted from the scanned images using Agilent's Feature Extraction software (ver. 9.1.3.1). All microarray processing was performed at the genomics core facility at the MIT BioMicro Center.

### Analysis of Motor Behaviors

The forelimb behaviors of the rats in the *Reach* groups were studied by analyzing the videos of their behavioral sessions, frame by frame (29.97 frames/s; 59.94 Hz after de-interlacing each frame into two fields), using VirtualDub ver. 1.9.11 (GNU General Public License release). To quantify the rat's performance, for every trial we documented both the number of reaches displayed before the pellet was grasped or knocked away, and whether the pellet was successfully retrieved. Pellet retrieval was defined to be successful if the pellet was moved from its original slot on the shelf to any position inside the slit or the behavioral box, or was passed directly to the rat's mouth. A single reach was defined to be any hand trajectory observed after the hand moved from the box to the shelf by crossing the slit-shelf border (represented by a thick black line in Fig. 1A), and before the hand came back into the box by crossing the same border again. Thus, within any single trial, the rat could exhibit multiple reaches before its hand came into contact with the pellet.

Motor skill learning across days was assessed using two measures. The first measure of motor skill learning quantified the quality of the motor behavior on successful trials. For some successful trials, the pellet was grasped but then dropped either to the inside of the slit or to the floor of the box before it was consumed by the rat. Thus, the percentage of successful trials with dropped pellets was calculated for each day as a measure of skill learning independent of task goal achievement (defined below). Since this measure refers to a post-grasping event, this measure was calculated across all slots together.

The second measure quantified the extent of achievement of the task goal: retrieval of food pellets. Task performance was measured by first counting the number of pellets retrieved in each session, and then dividing this number by the total number of reaches observed in all trials. The resulting value provides an empirical estimate of the probability of a successful retrieval for every reach displayed.

For this second measure of skill learning, we observed that most rats displayed between-session improvement in task performance over only a subset of the six slots, with different rats showing improvement over different subsets. For this reason, for every rat that received 12 days of training, we calculated task performance separately for the slots showing improvement (Learned Slots) and for the remaining slots not showing improvement (Not-Learned Slots). To determine whether each slot was a Learned or Not-Learned Slot, we first calculated task performance for each individual slot. This produced a noisy learning curve, presumably because the number of trials used in the statistic for each day was small (*n* = 20). We clarified the trend of the curve with a moving average filter (window width = 3 days), and then, computed the Pearson's correlation coefficient on the smoothed curve. Any slot showing a learning curve with a positive and significant (p<0.05) coefficient was placed in the “Learned” category; slots not meeting this criterion were placed in the “Not-Learned” category. After this determination, task performance was re-calculated for the Learned and Not-Learned Slots, respectively. Statistical significance of the difference in task goal achievement between the first and last five days of training was assessed by applying ANOVA to the original un-smoothened data.

The time course of task goal achievement of each rat was characterized by fitting the learning curve derived from the Learned Slots onto an exponential and a sigmoid function, respectively. The quality of these two regressions were then compared through their *R* ^2^ values. The exponential function used for learning curve fitting is given by the equation
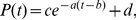
(1)


where *t* is training day, *P*(*t*) is performance at day *t*, *a* and *b* are unconstrained curve-fitting parameters, *c*<0, and 0<*d<*1. The sigmoid function we used is given by the expression 
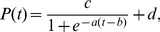
(2)


where *a*, *b*, *c*, and *d* are non-negative parameters subject to the following boundary conditions: *a* ≤ 10, *b* ≤ 12, *c* ≤ 1, and *d* ≤ 1. For every sigmoid fit, we also quantified the time when performance started to improve by calculating the time point on the regression curve at which 10% of the peak performance is achieved (*t*_10%-max_), given by the formula 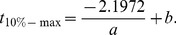
(3)


All curve fitting analyses were accomplished using the curve fitting toolbox of Matlab (ver. 7.11.0). Parameters were estimated by nonlinear least squares (trust-region algorithm).

### Analysis of Microarray Data

Gene expression values of each gene were presented as ratios between signal intensities derived from cy5 (labeling samples from the *Reach*- or *Sham-*rats) and those from cy3 (labeling the reference sample from the naïve rat). All probes whose array signals were deemed marginally acceptable or absent by Agilent's Feature Extraction software were omitted from further analysis. Standard Lowess normalization, background subtraction, and outlier exclusion were performed on the array data per the software's default settings.

The identification of differentially expressed genes was divided into two steps. In the first step, we sought to find genes whose differential expression at 5 or 12 days may specifically be attributed to the learning of the reach-and-grasp skill rather than to the execution of the task contingency related to trial initiation, or just to the passage of time. This isolation was accomplished by subtracting the list of genes differentially expressed in the *Sham* groups from that in the *Reach* groups, performed as follows. Using GeneSpring GX11 (Agilent), we compared gene expression between the *0-day, 5-day-Reach* and *12-day-Reach* groups, and then, between the *0-day, 5-day-Sham* and *12-day-Sham* groups. In each of these three-group comparisons, the Kruskal-Wallis test was used to evaluate the hypothesis that the mean expression of one group was different from those of the other two. In other words, the null hypothesis for each comparison is that the mean gene expressions of the *0-day*, *5-day* and *12-day* groups were the same. The set of differentially expressed genes identified in each comparison (p<0.05) was further filtered by the criterion that the between-group difference had to be greater than 1.5-fold in at least one of the three group-pairs. Subtracting the resulting *Sham*-group gene list from the *Reach*-group list then yielded a list of genes whose differential expression in one or both of the *Reach* groups, with respect to *0-day,* may specifically be related to forelimb skill learning.

The above subtraction isolated probes differentially expressed in the *Reach* but not in the *Sham* groups. For completeness, we similarly identified probes differentially expressed in the *Sham* but not in the *Reach* groups. Presumably these *Sham*-related genes may be related to any behavioral or other peculiarities specific to the *Sham* animals (see Discussion).

The second step of our analysis was aimed at isolating, from the lists we obtained in the first step, probes with similar dynamics of differential expression from Day 5 to Day 12. For each of these two days and for every probe in the list of genes differentially expressed in *Reach* groups, we computed the difference in average expression between the *Reach* and *Sham* groups, so that the resulting difference represents the probe's differential expression, with respect to the *Sham* group, at Day 5 or Day 12. A scatter plot of the Day-12 difference values against the Day-5 difference values of all modulated probes allowed us to visually identify distinct data-point clusters on the graph; each cluster would then comprise probes having similar relationships between their differential expressions at 5 and 12 days. This analysis was performed using custom software written in Matlab.

For the list of genes differentially expressed in the *Sham* groups obtained from step one, a similar difference in average expression between the *Sham* and *Reach* groups was calculated with differential expression defined with respect to the *Reach* group instead.

Functional categorization of the list of genes regulated at each day was accomplished using the functional annotation tool of DAVID Bioinformatics Resources ver. 6.7 [17]. The annotation sources and databases employed included the Clusters of Orthologous Groups (COG), Swiss-Prot (SP), Protein Information Resources (PIR), Uniprot Sequence Feature (UP), Gene Ontology (GO), Protein Analysis Through Evolutionary Relationships (PANTHER), Pubmed ID, InterPro, and KEGG Pathway. All clustering was performed with classification stringency set at medium. The importance of each gene cluster was evaluated by an enrichment score computed with the rat genome set as the gene population background.

### Gene Expression Assays by qPCR

We further examined the expression of 18 genes, all found to be up-regulated at Day 5 by our array analysis, using quantitative reverse transcription polymerase chain reaction (qPCR). The total RNA samples used for array hybridizations in four of the five conditions (*0-day, 5-day-Reach*, *5-day-Sham*, and *12-day-Reach*) were first reverse transcribed using the High-Capacity cDNA Reverse Transcription Kit of Applied Biosystems (Carlsbad, CA). Gene amplification was achieved in 20 µL-qPCR reactions containing a mixture of the cDNA sample with the gene's forward and reverse primers (200 nM for each) and the SYBR Green Supermix of Bio-rad (Hercules, CA), performed in triplicate for each sample and carried out in a thermal cycler (iCycler, Bio-rad). The primer pair for each gene was designed using the online Primer-BLAST resource provided by the National Center for Biotechnology Information, USA, and their amplification efficiency was characterized by the standard C_T_ curve obtained by a 5-fold dilution series of a sample isolated from a naïve rat (Table 1). Before assaying the expression of our genes of interest, we assessed the stability in expression of six genes, including glyceraldehyde-3-phosphate dehydrogenase, 18S subunit ribosomal RNA, cyclophilin A, beta actin (*Actb*), tyrosine 3-monooxygenase/tryptophan 5-monooxygenase activation protein, zeta polypeptide (*Ywhaz*) and ribosomal protein L13A, that may potentially serve as internal reference genes for normalization of C_T_ values [18], across three conditions (*0-day, 5-day-Reach* and *5-day-Sham*). For each of these genes, the C_T_ values obtained were regressed against the RNA concentration of the samples (determined by the absorbance at 260 nm), and the two genes with the highest correlation coefficients – *Actb* and *Ywhaz* – were selected to be the reference genes for subsequent qPCR experiments. The sample C_T_ values obtained were then normalized to each of the two references. Statistical significance of the difference between the normalized C_T_ of the *5-day-Reach* samples and those of the other samples was assessed using the Kruskal-Wallis test (α = 0.05). Fold differences in gene expression between conditions were calculated using the C_T_ values, normalized to whichever reference gene that yielded a smaller p-value in the above statistical test, using the formula, fold change = 2^-*Δ*(*Δ*Ct)^, where δ(δCt) is the change of normalized C_T_ (δCt) between conditions.

**Table 1:** Forward and reverse primers used for genes validated with qPCR.

Gene Symbol	Accession No.	Forward (+) and reverse (−) primers	Position on Gene Sequence	Efficiency (%)
Actb (reference 1)	NM_031144.2	(+)TGTCACCAACTGGGACGATA	306–325	97.3
		(−)GGGGTGTTGAAGGTCTCAAA	470–451	
Adcy1	NM_001107239.1	(+)CTTCGGGCTCGTGGTGGCTG	414–433	102.3
		(−)CCAGAGACGTGGGCGCTTGG	489–470	
Cask	NM_022184	(+)GCGGGATCGTTATGCCTACA	1191–1210	93.2
		(−)TGAGGAGGTAGGGTCTTCGG	1371–1352	
Ctnnd1	NM_001107740.1	(+)GGGCTACCGGGCACCCAGTA	1111–1130	108.1
		(−)CGTCGAGGGTCCGAGGGTGT	1320–1301	
Ephb2	NM_001127319	(+)CCGCCGTGGAAGAAACACT	50–68	107.7
		(−)TACAGTCACGCACCGAGAAC	292–273	
Fgf14	NM_022223.2	(+)GCCAGCGGCTTGATCCGTCA	16–35	92.6
		(−)GCAAAGCCCGCGGTTCTTGC	120–101	
Fgf2	NM_019305.2	(+)GGCGGCTTCTTCCTGCGCAT	638–657	98.8
		(−)AGCCAGGTACCGGTTCGCAC	781–762	
Fgfr3	NM_053429.1	(+)AGTGTTCTGCGTGGCGGTCG	24–43	96.4
		(−)ACAGCACACGCCGGGTTAGC	349–330	
Frs2	NM_001108097.3	(+)GGTCGGGTCGCGGAGAGAGT	79–98	107.2
		(−)AGCCATTTCGTCGGCGCGAA	158–139	
Map2k7	NM_001025425.1	(+)CTGAGCGCATTGACCCGCCA	857–876	100.3
		(−)CCCAGGCTCCACACATCGGC	926–907	
Mapk14	NM_031020.2	(+)GGCCCACGTTCTACCGGCAG	317–336	100.4
		(−)GCAGCACACACCGAGCCGTA	425–406	
Nsf	NM_021748	(+)GCATCGGCACAATGACCATC	276–295	114.8
		(−)TGTCGGTGTCGTAAGGGTTG	350–331	
Prkar2a	NM_019264.1	(+)GCCGGCATGAGCCACATCCA	108–127	90.6
		(−)GCTGGACTCCTGCGCGTGAA	305–286	
Prkci	NM_032059.1	(+)ATTTACCGCAGAGGGGCGCG	511–530	103.2
		(−)CGGTGGCAAAGAATGCCGCC	732–713	
Rab11fip4	NM_001107023.1	(+)AGCCCTTGCCCTGACGACGA	739–758	100.7
		(−)AGCCGGTCAGACATGCGCTG	1430–1411	
Stx16	ENSRNOT00000007054*	(+)GGCTACTGCGGAATGTGGT	473–491	106.2
		(−)GGGTGTCGAAGAAATGCTGC	607–588	
Syt1	AJ617619	(+)CAACCAACATCCGCAGTCAGA	10–30	92.8
		(−)TCATGTTAATGGCGTTCTTTCTTCA	111–87	
Tmod2	NM_031613	(+)GACGAGGACGAGCTTCTTGG	103–122	107.3
		(−)GGGTCTGGTCTTTCTGTCGG	226–207	
Ywhaz (reference 2)	BC094305.1	(+)TTGAGCAGAAGACGGAAGGT	560–579	92.1
		(−)GAAGCATTGGGGATCAAGAA	695–676	
Zfp238	NM_022678.1	(+)CCCTCAAGCGCCACGAGAGG	1271–1290	91.7
		(−)AACCTGCGCTCGCACCACTT	1424–1405	

* : The complete sequence of *stx16* (syntaxin 16, fragment) can be accessed through the Treefam database with the identification number TF314090.

## Results

### Rat Forelimb Task Performance Exhibited a Sigmoidal Learning Curve

We seek to identify genes differentially expressed in the motor cortex during the acquisition of a motor skill by training rats to reach and grasp for food pellets, using the preferred forelimb, from randomized locations in the workspace. To assess the effects of training on forelimb motor behavior with our behavioral paradigm, we first examined the change in the quality of the reach-and-grasp behavior over time. During the initial days of training, in many of the successful trials, after the pellet was grasped and retrieved it was subsequently dropped either into the slit or onto the floor of the box before it was picked up again by the rat with its tongue. This observation prompted us to calculate, for each day, the percentage of successful trials with dropped pellets as a measure of learning. The lower the percentage, the higher the number of trials in which the pellet was passed directly from the slot to the mouth.

**Figure 2:**
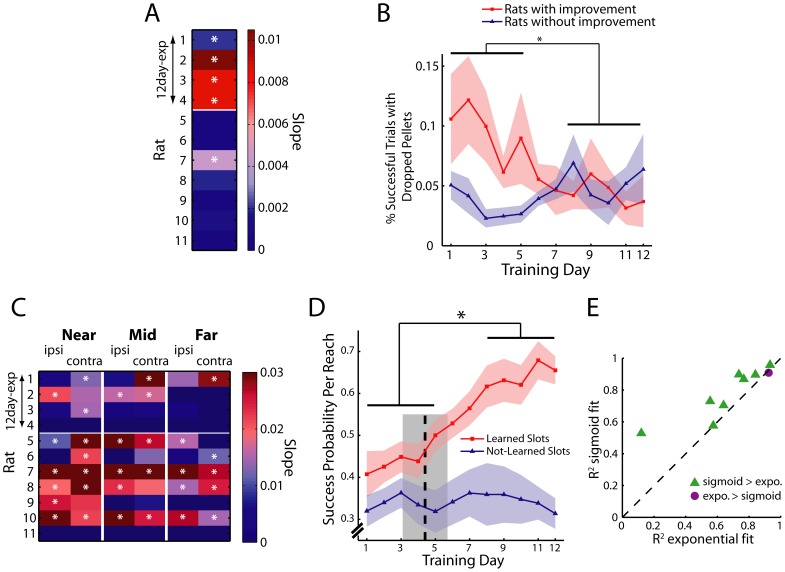
Improvement in task goal achievement exhibited a sigmoid time course. *A*, Eleven rats (rats 1–4 in *12-day-Reach* group) were trained for 12 days to reach and grasp pellets from six different locations in the workspace, including slots ipsilateral (ipsi) and contralateral (contra) to the rat's preferred side, at the Near, Mid, and Far levels (Fig. 1A), respectively. The first measure of motor learning we used indicates the quality of reaching by quantifying the percentage of successful trials in which the pellet was dropped either to the inside of the slit or to the box's floor. Different rats showed different rates of improvement (blue, low rate of decrease; red, high rate) with 5 of the 11 rats showing a statistically significant decrease (*, Pearson's correlation coefficient<0, p<0.05). The top four rows correspond to data from rats included in the microarray analysis. *B,* For the 5 rats showing across-day improvement in movement quality, there was a clear decrease in the percentage of successful trials with dropped pellets over time (red, mean±SE). The percentage values of the first five days were greater than those of the last five days for these rats (*, p<0.05, ANOVA) but not for the other rats (blue). *C,* As a second measure of skill learning, we quantified the degree of task goal achievement across days. We examined whether there was across-day increase in the probability of successful retrieval per reach for each individual slot by linearly regressing the learning curve for each slot against time. Different rats exhibited performance improvement at different rates at different slots (blue, low improvement rate; red, high improvement rate) with different subsets of slots showing a significant increase in the success probability (*, Pearson's correlation coefficient>0, p<0.05; “Learned” slot). The top four rows correspond to data from rats included in the microarray analysis. *D,* The probability of successful retrieval per reach was calculated over the slots showing significant improvement (Learned Slots; red, mean±SE) and the other remaining slots showing no improvement (Not-Learned Slots; blue), respectively. The learning curve for the Learned Slots exhibited a sigmoid time course with the success probability starting to increase at Day 4.4±1.3 (t_10%-max_, black dotted line; mean±SE). For the Learned Slots, the probability values of the first 5 days were also significantly lower than those of the last 5 days (*, p<0.05, ANOVA). *E,* The learning curve from the Learned Slots of every rat was regressed onto a sigmoid and an exponential function, respectively. When the regression R^2^ values of the sigmoid fit were plotted against those of the exponential fit, all but one data point lay above the identify line (black dotted line) (green triangle, sigmoid>exponential; purple circle, exponential>sigmoid).

Of the 11 rats receiving 12 days of training, 5 showed a significant improvement in movement quality over time (Pearson's correlation coefficient<0, p<0.05) (Fig. 2A, *). The learning curve derived from these rats showed a decrease in this percentage from Day 1 to 6, and reaching an asymptotic minimum at Day 7 (Fig. 2B, red). Results of ANOVA tests confirmed that the difference between the percentage values of the first five days and those of the last five days was statistically significant for the rats showing improvement in movement quality (Fig. 2B, red; F(1,49) = 10.3, p<0.01) but not for the other rats (Fig. 2B, blue; F(1,59) = 3.7, p>0.05).

A second measure of motor skill learning we used quantifies achievement of the task goal (i.e., pellet retrieval) across days using the empirical probability of a successful retrieval for each reach, in the rats receiving 12 days of training. We found that most rats displayed across-day improvement in task performance over only a subset of the six slots, with different rats showing improvement over different subsets. In particular, 2 rats showed no improvement in any slot; 7 rats, improvement in a subset of slots; and 2 rats, improvement in all six slots (Fig. 2C). This observation prompted us to derive separate learning curves for the Learned Slots and Not-Learned Slots, respectively. The learning curve for the Not-Learned Slots (Fig. 2D, blue) showed no increase in success probability over time. In contrast, for the Learned Slots (Fig. 2D, red), performance increased slowly from Day 1 to 4, then sharply from Day 4 to 8, and then more slowly from Day 8 to 12. Results of ANOVA tests confirmed that the difference between the success probability values of the first five days and those of the last five days was statistically significant for the Learned Slots (F(1,89) = 51.9, p<0.01), but not for the Not-Learned Slots (F(1,89) = 0.08, p>0.05).

The shape of the learning curve for the Learned Slots (Fig. 2D, red) suggests that the time course of task performance was closer to a sigmoid pattern in which performance increases notably only after an initial phase with small improvement. Such a learning time course stands in contrast to the classic power-law or exponential performance observed in other motor learning studies (e.g., [1], [19]). When we fit the learning curve of each rat to both an exponential (eq. 1) and a sigmoid (eq. 2) function, a sigmoid fit to the learning curve yielded a higher regression R^2^ than an exponential fit in all but 1 rat (Fig. 2E). Thus, in our behavioral paradigm the time course of learning, as quantified by task goal achievement, is better described as a sigmoid performance. To objectively determine the time when performance started to increase, we calculated the time point on the sigmoid fit at which 10% of the maximum performance was attained (t_10%-max_; eq. 3). This was found to be at Day 4.40±1.31 (mean±SE; Fig. 2D, black dotted line).

**Figure 3:**
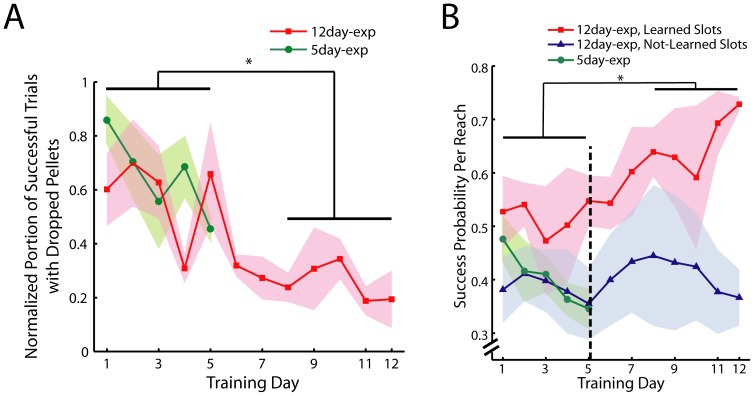
Rats used for microarray analysis exhibited motor behaviors similar to the behaviors of the whole rat group. *A,* The quality of the reaching behavior of the 4 rats in the *12-day-Reach* group (red, mean±SE) showed much improvement over time with the percentage of successful trials with dropped pellets of Day 8 to 12 being clearly lower than that of Day 1 to 5 (*, p<0.05, ANOVA). Similarly, a decreasing trend of this measure of learning was also observed in the *5-day-Reach* group (green). In this figure we have normalized the learning curve of every rat to its maximum value to account for inter-individual variability in initial performance. *B,* The probability of successful pellet retrieval per reach for the rats in the *12-day-Reach* group over the Learned (red, mean±SE) and Not-Learned (blue) Slots. Similar to the behavioral trend of the whole rat group (Fig. 2D), the learning curve for the Learned Slots exhibited a sigmoid time course, with performance starting to increase at Day 5.1±3.2 (t_10%-max_, black dotted line, mean±SE), and with the probability values of Day 1 to 5 smaller than those of Day 8 to 12 (*, p<0.05, ANOVA). The success probability values for the *5-day-Reach* group (N = 4; green; all slots) were also not statistically different from the values for the *12-day-Reach* group over the first 5 days (p>0.05, ANOVA).

Four of the eleven rats receiving 12 days of training were selected for further microarray analysis (*12-day-Reach* group). They were selected because they displayed robust improvement in movement quality (Fig. 2A), and because the RNAs extracted from their brains were of high quality (RNA Integrity Number≥8.5). Three of the four rats in this group also showed good improvement in task performance over 1 to 3 slots (Fig. 2C). We additionally verified that the behavioral time course exhibited by these four rats was not different from the trend demonstrated for the whole group. Specifically, the quality of reaching, quantified by our first measure of motor learning, improved from the first five days to the last five days of training (Fig. 3A, red; F(1,39) = 8.5, p<0.01). Similarly, for these four rats the success probability per reach for the Learned Slots also assumed a sigmoid time course (Fig. 3B, red), and the probability values of the last five days were significantly higher than those of the first five days (F(1,29) = 9.42, p<0.01). The probability value started to increase at around Day 4 to 5 with the t_10%-max_ of the sigmoid fit determined to be at Day 5.07±3.19 (Fig. 3B, black dotted line).

Overall, our two measures of motor learning suggest the following time course of motor skill acquisition in our behavioral paradigm. From Day 1 to around Day 4 to 5, there was little improvement in task goal achievement (Figs. 2D, 3B), but during the same period the rat quickly mastered the skill of passing the pellet directly to the mouth once it was grasped (Figs. 2B, 3A). This improvement in movement quality probably reflects an initial process of trajectory optimization for the task at hand that occurs independently of the process that drives increase in task goal achievement. The probability of successful retrieval started to increase at around Day 5 as suggested by the t_10%-max_ values determined from the sigmoid fit. We therefore decided to harvest motor cortical tissues after 5 days of training from another group of rats (*5-day-Reach* group) to examine how the transcriptome may have changed at the time when task goal achievement had not changed much from initial performance, but was about to improve substantially.

### Motor Behaviors of the *5-day-* and *12-day-Reach* Groups were Similar

The motor behaviors of the *5-day-Reach* rats were overall very similar to those of the *12-day-Reach* rats exhibited during the first five training days. For the movement quality measure of learning, the raw percentage values (Day 1 to 5) for the *5-day-Reach* rats were found to be higher than those for the *12-day-Reach* values (F(1,39) = 7.8, p<0.05). However, after normalizing the learning curves of both groups to their maximum, not only was there no significant difference between the values of the two groups (F = 0.67, p>0.05), but the rate of decrease on the learning curve from Day 1 to 5, as determined by the slope from linear regression, was also not different between the two groups (Fig. 3A, green versus red; p>0.05). Thus, the inter-group difference in movement quality before normalization reflects inter-individual variability in the initial performance on Day 1 rather than difference in the trend of improvement over time. For the task performance measure of motor learning, the success probability values (Day 1 to 5) of the *5-day-Reach* group (all slots) (Fig. 3B, green) were also not significantly different from those of the *12-day-Reach* group for the same time period (F(1,54) = 1.43, p>0.05).

The overall similarity between the behavioral trends of the *5-day-Reach* and *12-day-Reach* groups argues that any changes in the transcriptome in either group, as compared with *Sham* groups, reflect gene expression dynamics in the motor cortex during skill learning rather than any inter-group difference in learning pattern.

### Successful Pellet Retrieval Demanded Strategic Hand Placement

**Figure 4:**
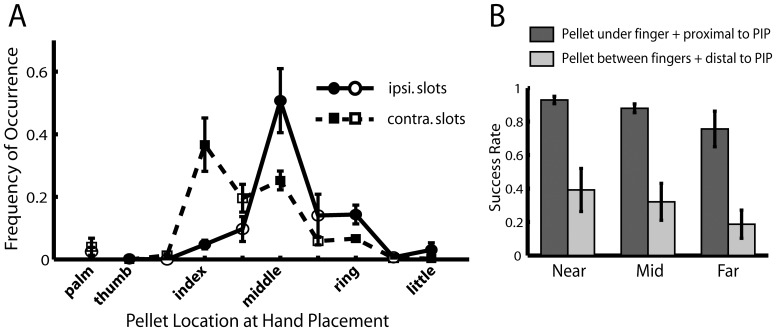
Successful pellet retrieval demanded strategic hand placement. *A*, The frequency of use of different digits in successful trials for ipsilateral and contralateral slots. In the successful trials, immediately before grasping, each rat positioned its paw so that the pellet was directly under one of the digits, in between two adjacent digits, or under the palm of the hand. The graph shows the frequency of occurrence for the above possibilities (filled symbols: under a digit; unfilled symbols: between digits or under palm) within the successful trials for the ipsilateral (circles connected by solid line) and contralateral (squares connected by dotted line) slots (mean±SEM), across the animals in the *12-day-Reach* group (average over Day 1, 5, and 12). The two distributions were clearly separated with different means (Slot×Digit interaction, F(9,60) = 6.8, p<0.01) with the ipsilateral distribution peaking at the middle finger, and the contralateral, at the index finger. This suggests that grasping from these two different sets of slots involve the use of different digit sets. *B*, Strategies employed for slots at different distances from the rat. We calculated the rate of successful retrieval when, at the time of maximal forelimb extension, the pellet was directly under a finger (instead of between fingers) and was proximal in position to the PIP joint (dark grey bars), and when neither of these strategies was employed (light grey bars), over the near, mid, and far slots, respectively (mean±SEM). For all three slot sets, the success rate when the strategies were employed was clearly higher than when neither was employed (p<0.01).

To understand exactly what motor strategies the rats had learned to achieve a higher task performance over time in multiple slots, we determined whether successful pellet retrieval from different slots demanded the same or different motor actions. A frame-by-frame analysis of the video record of the movement trials (*12-day-Reach* group) revealed that grasping from the ipsilateral and contralateral slots promoted the use of different digits. In the successful trials with ipsilateral targets, immediately before grasping the rat placed its hand over the pellet mostly under the middle finger whereas in successful trials with contralateral targets, mostly under the index finger (Fig. 4A). The clearly separated distributions of digit use for the ipsilateral and contralateral targets (slot×digit interaction, F(9,60) = 6.8, p<0.01) suggests that pellets at these two sets of slots were grasped by the hand through the flexion motion of different digits.

Slots at the near, mid, and far levels also demanded different trajectories for retrieval success. We observed in our video records that in many successful trials, at the point when the arm was maximally extended before the hand contacted the pellet, the pellet was consistently proximal in position to the proximal interphalangeal (PIP) joint of the finger covering the pellet, suggesting that the further away the pellet is from the rat, the more the forelimb has to extend to ensure success. Indeed, for all of near, mid, and far slots, the success rate for trials in which the pellet was both directly under a finger and proximal to the PIP joint when the paw contacted the pellet was much higher than when neither strategy was employed (Fig. 4B; main effect of strategy, F(1,18) = 41.8, p<0.01).

These behavioral analyses verify that achieving the task goal in our behavioral paradigm demanded a nontrivial skill of placing the hand strategically relative to the pellet's position. The variable position of the pellet across trials forced the rat to adjust its motor plan before every trial according to its perception of where the pellet was placed. Any increase in the success rate over time in more than one slot thus constituted either the simultaneous learning of multiple visuomotor associations between different slots and different motions, or the learning of a more general strategy of motor adjustment based on the perceived task goal.

### Microarray Data had Consistent Quality Across Groups

**Figure 5:**
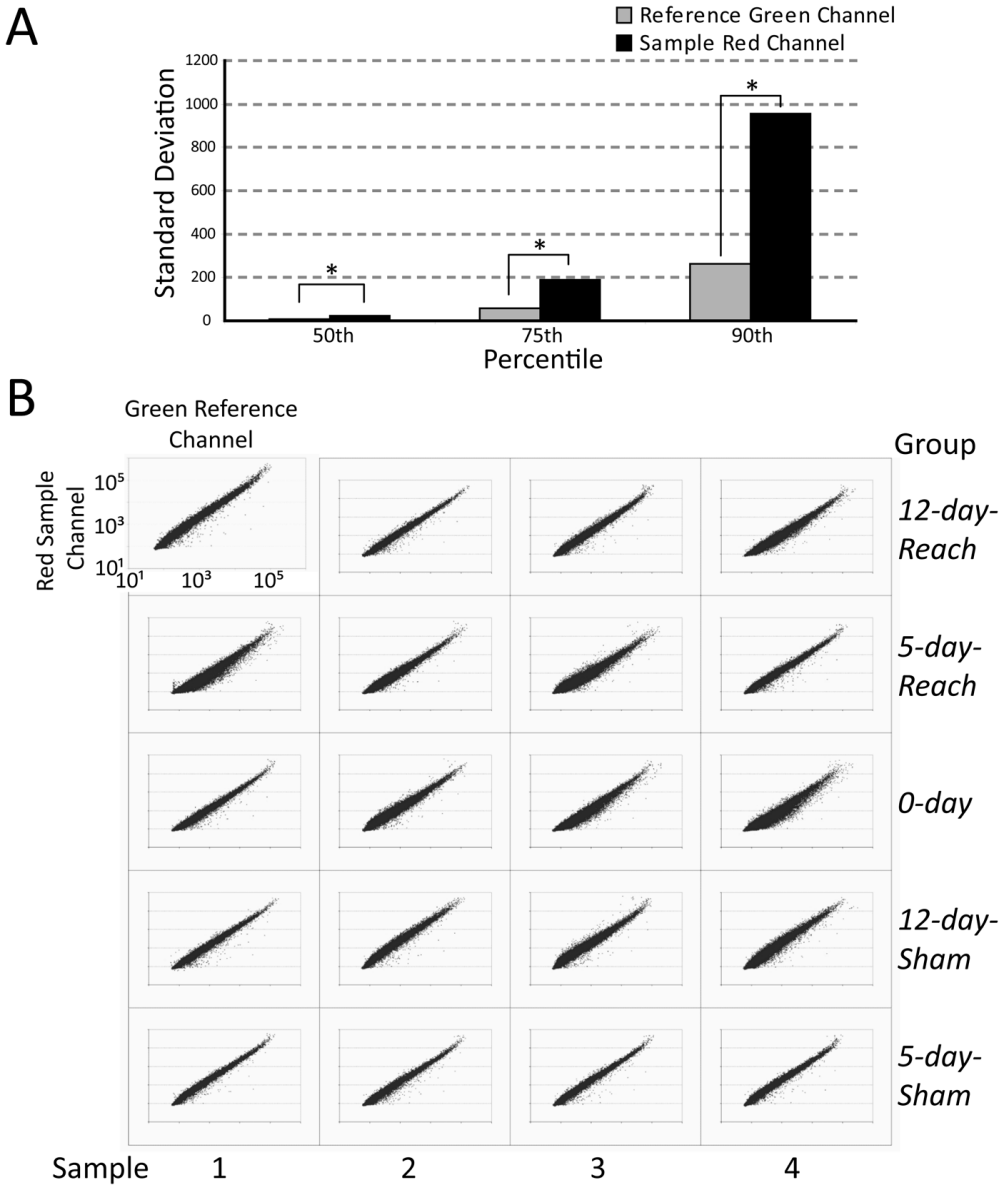
Microarrays had consistent data quality across groups. *A,* We verified that in our 2-color hybridizations, most of the inter-group variation in gene expression was due to variability of the sample channel (red) but not the reference channel (green). We computed the standard deviation, across the 20 arrays, of the raw signal intensity of the sample red channel (black) and the reference green channel (light grey), respectively, at three percentiles of the signal distribution. For all tested percentiles, the standard deviation from the sample channel was clearly much higher than that from the reference channel (F-test on variances, p<0.01). *B,* We further validated that the hybridization quality of the 20 arrays was consistent across experimental groups. We show here a scatter plot of the raw signal intensity (log scale) of the red sample channel against that of the green reference channel for each microarray (N = 41,012). As can be seen, the overall ratio of signal intensities between the two channels was very reproducible across arrays, with the slope of the linear regression on these data not significantly different across the five groups (p>0.05, ANOVA). Thus, any differential gene expression we identified is unlikely to be just due to fluctuation of signal intensity in one of the two channels as a result of inconsistent hybridization quality across arrays. Note that the scatter plot of the upper-left panel is enlarged relative to the others for the graphical purpose of clearly depicting the axis labels.

Before we analyzed the differential expression of the probes on our microarrays, we evaluated both the overall quality of our processing as well as the consistency of data quality across groups. In our experimental design, the transcriptome of each animal was hybridized to a single microarray. The Agilent arrays we used employed a two-color design for which the gene expression value of each probe was presented as a ratio between the signal derived from cy5, or the "red" channel (labeling samples from the trained *Reach*- or *Sham*-rats), and that from cy3, or the "green" channel (labeling a reference sample from a naïve rat). It is thus important to verify that any observed inter-array variability of the gene expression data owes its origin to signal variability of the sample red channel rather than that of the reference green channel. We confirmed this by computing the standard deviation of the signal intensity of each channel, across the 20 microarrays, at three different percentiles of the raw-data signal distribution. At all tested percentiles, the standard deviation of the red (sample) channel was clearly much higher than that of the green (reference) channel (Fig. 5A), and these differences were statistically significant (F-test on variances, p<0.01). Therefore any inter-array variability of gene expression should have a biological origin.

We further validated that the quality of the two-color hybridization experiment was consistent across experimental groups by examining the overall ratio of signal intensities between the two channels. A scatter plot of the raw signals from the red channel versus those from the green channel for each of the 20 arrays revealed that this signal-intensity ratio was very reproducible across arrays (Fig. 5B) with the slope of the linear regression on the data not being significantly different across the different experimental groups (ANOVA, p>0.05). Thus, the probes found to be differentially expressed in our analysis below were very unlikely to be just an artifact arising from inconsistent data quality across groups.

### Most Genes were Differentially Expressed Immediately Preceding Task Improvement

**Figure 6:**
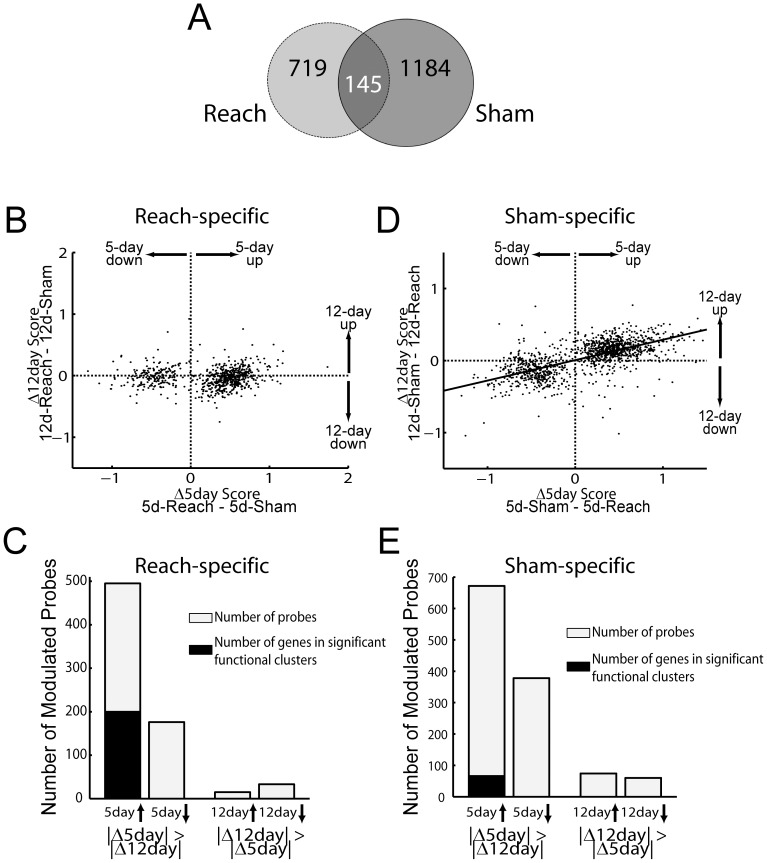
Most of the modulated probes in the *Reach* groups were differentially expressed at 5, but not 12, days. *A*, Isolating differentially expressed probes related to motor skill learning. The Venn diagram shows the degree of intersection between the set of modulated probes (Kruskal-Wallis test; p<0.05) in the *0-day* vs *5-day-Reach* vs *12-day-Reach* comparison (light grey circle), and that in the *0-day* vs *5-day-Sham* vs *12-day-Sham* comparison (dark grey circle). The 1184 probes specific to the *Sham* groups probably reflect gene expression changes that occurred as a result of the execution of the task contingency, the passage of time, and other factors, while the 719 modulated probes specific to the *Reach* groups are most likely specifically related to processes underlying motor skill learning. *B*, Dynamics of differential gene expression from 5 to 12 days for the modulated probes specific to the *Reach* groups. For every modulated probe, the extent of differential expression at 5 and 12 days (Δ5day, x-axis; and Δ12day, y-axis) were calculated by subtracting the mean expression of the *Sham* group of each day from that of the *Reach* group. A scatter plot of the 719 data points shown here revealed two distinct groups of probes clustering around the positive and negative Δ5day-axes with relatively small amplitudes along the Δ12day-axis, suggesting that most of the modulated probes were differentially expressed at 5 days but not at 12 days. *C*, Dynamic types of differential expression for the *Reach*-specific probes. Each modulated probe was grouped into one of the four dynamic types, depending whether |Δ5day| was larger (bars 1 and 2) or smaller (bars 3 and 4) than |Δ12day|, and whether the day with the larger magnitude of differential expression was an up- (bars 1 and 3) or down-regulation (bars 2 and 4). Most of the probes were grouped into the first and second types indicating a regulation at 5 days. The black bar indicates the number of genes up-regulated at Day 5 categorized into gene clusters in subsequent DAVID functional analysis. *D*, Dynamics of differential gene expression from 5 to 12 days for the modulated probes specific to the *Sham* groups. Unlike the *Reach*-specific genes shown in *B*, Δ5day and Δ12day for these probes showed a positive, significant correlation (p<0.05). It is therefore possible that these *Sham*-specific modulations are driven by a process with a dynamics very different from that underlying forelimb skill learning. *E*, Dynamic types of differential expression for the *Sham*-specific probes. Notice that in the set of probes up-regulated at Day 5, the number of probes categorized into functional clusters by DAVID (black bar) was much smaller than that for the *Reach*-specific probes, shown in *C*.

To identify differentially expressed genes in the motor cortex after 5 or 12 days of training, we hybridized the RNAs isolated from 5 groups of rats (*0-day, 5-day-Reach*, *5-day-Sham*, *12-day-Reach*, and *12-day-Sham*) onto Agilent whole-rat genome microarrays. The Kruskal-Wallis test (α = 0.05) was applied over gene expression data of every probe from the *0-day*, *5-day-Reach* and *12-day-Reach* groups to isolate genes differentially regulated in one or both of the *Reach* groups as compared with *0-day,* and similarly, over data from *0-day, 5-day-Sham* and *12-day-Sham* groups to isolate genes regulated in one or both of the *Sham* groups. Out of the 41,012 probes on the microarray, we found a total of 864 differentially expressed probes from the *Reach-*group comparison, and 1,328 probes from the *Sham*-group comparison. We reasoned that the list of modulated probes for the *Sham* groups reflects gene expression changes that occurred as a result of the execution of the task contingency, the passage of time, and/or other behavioral peculiarities specific to the *Sham* animals (see Discussion for interpretations). A list of probes related specifically to forelimb skill learning of the *Reach* groups was then obtained by subtracting the *Sham* probe list from the *Reach* list, which yielded 719 probes (491 annotated with gene symbols, corresponding to 466 unique genes) present only in the *Reach* probe list but not in the *Sham* probe list (Fig. 6A, left circle). For completeness, we similarly obtained a list of *Sham*-specific probes by subtracting the *Reach* probe list from the *Sham* list, which yielded 1184 probes (538 annotated with gene symbols, 518 unique genes) (Fig. 6A, right circle).

The temporal expression profiles of the modulated genes identified above were then characterized by examining whether there were discernable relationships between the expression intensities at Day 5 and Day 12, for both the *Reach*-specific and *Sham*-specific probe lists. For the *Reach*-specific list, the extent of differential gene expression of each probe at each day was calculated by subtracting the mean expression value of the *Sham* group from that of the *Reach* group (denoted by Δ5day and Δ12day for values at Day 5 and Day 12, respectively). A positive Δ5day corresponds to an up-regulation of expression at Day 5; a negative Δ5day, down-regulation; and similarly for positive and negative Δ12day values. A scatter plot of Δ12day (Fig. 6B, y-axis) against Δ5day (x-axis) revealed two groups of probes clustered around the positive and negative x-axis, containing probes up-regulated at 5 days and probes down-regulated at 5 days, respectively. Furthermore, the Δ12day- and Δ5day-scores were uncorrelated (*r* = 0.056, p>0.05; slope = 0.018). The fact that the data points within these clusters had small Δ12day values suggests that most of the modulated probes were differentially regulated at Day 5 but not at Day 12.

**Figure 7:**
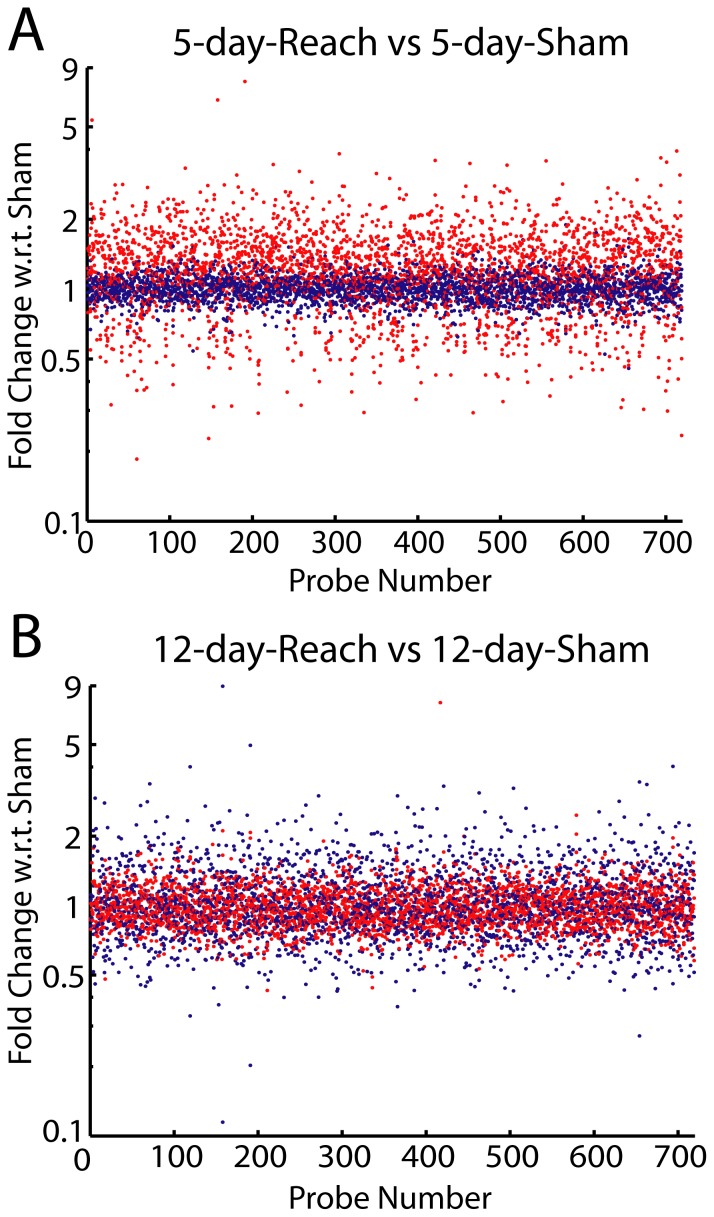
Most *Reach*-specific probes were up-regulated at Day 5 but not at Day 12. A scatter plot of the gene expression intensities of the *Reach* (red) and *Sham* (blue) groups, at Day 5 (*A*) and Day 12 (*B*), for all *Reach*-specific probes (N = 719). At Day 5, the *Reach* data points clearly lay above the *Sham* data points (Kruskal-Wallis, p<10^-4^) whereas at Day 12, the two sets of data points overlapped each other (p>0.1). Note that in *A*, the data of all *Reach*-specific probes, including both up- and down-regulated probes, are plotted.

We further classified each modulated probe into one of the four dynamic types, depending on whether the magnitude of differential expression at Day 5 (|Δ5day|) was larger or smaller than that at Day 12 (|Δ12day|), and whether the day with the larger magnitude of differential expression was an up- or down-regulation. The vast majority of the 719 probes were primarily modulated at Day 5 with |Δ5day| larger than |Δ12day| (Fig. 6C, 93%). Within these probes, there were many more up-regulated probes (69%) than down-regulated probes (24%). Thus, there was prominent up-regulation of gene expression after 5 days of training, a point at which task performance was about to improve, than after 12 days of training when performance had already reached its peak (Figs. 2D, 3B). This conclusion can be readily visualized with a scatter plot of the expression data of the *Reach* and *Sham* animals. At Day 5 (Fig. 7A), the *Reach* data cluster (red) was clearly above the *Sham* cluster (blue) (Kruskal-Wallis, p<0.01); at Day 12 (Fig. 7B), by contrast, the two clusters overlapped completely (p>0.05).

We then performed a similar analysis for the modulated probes present in the *Sham* list but not in the *Reach* list. Differential expression of each probe at each day was calculated by subtracting the mean expression value of the *Reach* group from that of the *Sham* group. For this *Sham*-specific list, we found that Δ5day and Δ12day were positively correlated (*r* = 0.66, p<0.01; slope = 0.28) (Fig. 6D), which differs from the lack of correlation in the *Reach*-specific list (Fig. 6B). This contrast suggests that differential expression of the *Sham*-specific probes followed a time course different from that of the *Reach*-specific probes. However, since the slope of this correlation was small, most probes were still primarily up- or down-regulated at Day 5 (89%), with |Δ5day| greater than |Δ12day| (Fig. 6E).

### Modulated *Reach*-specific Genes were Categorized into Synapse- and Growth Factor-related Functional Clusters

To gain functional insight into the list of probes modulated at Day 5, we selected those in the list annotated with gene symbols and grouped them into functional clusters using the DAVID bioinformatics resources [17]. The list of annotated probes primarily up-regulated at Day 5 in the *Reach*-specific list contained 339 unique genes. Using DAVID's medium clustering stringency, these genes were grouped by the algorithm into a total of 120 functional clusters, of which 13 were significantly enriched with genes of a particular biological process, component, or function (enrichment score>2.0; p<0.01) against the rat genome background. Together, these significant clusters comprised 200 genes (59% of all modulated probes with gene symbols).

**Figure 8:**
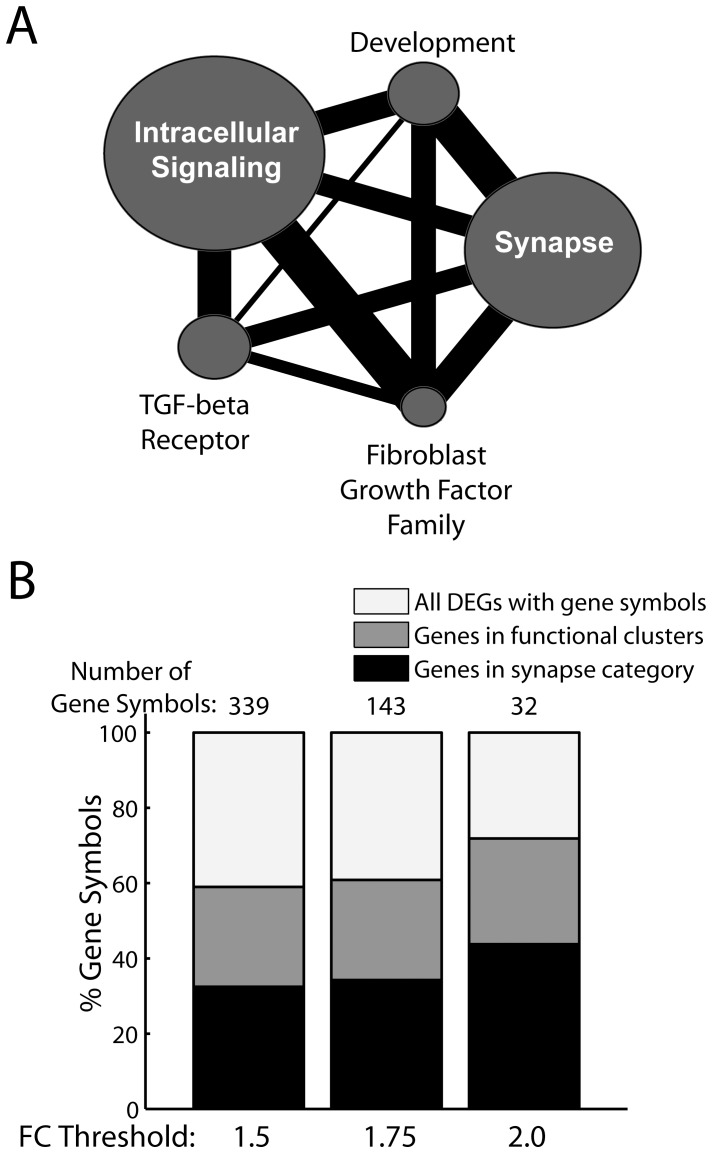
Differentially expressed genes were grouped into functional clusters related to the synapse, the fibroblast growth factor family, and other processes. *A,* The genes up-regulated at 5 days were grouped into 13 significantly enriched annotation clusters (p<0.01) using the DAVID bioinformatics resources. These clusters were then further condensed into 5 categories depending on the biological relationships between the clusters' annotation terms. Each of the five circles shown here represents one gene category. The area of the circle is directly proportional to the number of genes in that category. The thickness of the line connecting any two circles is proportional to the degree of overlap between the connected categories, indicated by the percentage of genes in the smaller of the two that are also present in the other. *B*, To estimate the effect of tightening the criterion for selecting differentially expressed genes on the rate of false positives, we successively increased our fold-change threshold from 1.5, to 1.75, and then 2.0 fold, and examined, at each threshold, the proportion of genes categorized into functionally enriched clusters. The percentages of genes remaining in any clusters (dark grey bars) and in the synapse category (black bars) were relatively unchanged as the fold-change threshold was increased. Assuming that the “true” proportion of "clusterable" genes is itself independent of the fold-change threshold, this observation implies that the false positive rate would probably not be much lower even with a more stringent fold-change threshold. DEG, differentially expressed genes.

Based on the genes and annotation terms represented in each cluster, we further condensed these 13 clusters into 5 categories of genes, each comprising one or more functionally related or similar clusters, so as to facilitate biological interpretation of the data (Fig. 8A and Table 2). The first category (Table 2) included 7 gene clusters related to the synapse, all potentially relevant to the regulation of synaptogenesis and synaptic plasticity: Cluster 2 contained many genes related to neurite outgrowth, regulation of dendritic spine morphology, and axonogenesis (examples of individual genes are given in Discussion); cluster 4 included genes related to cell adhesion, cell membrane trafficking, ion channels and other membrane components which may be necessary for the maturation of new synapses; cluster 10 was explicitly linked to synaptogenesis by its annotation terms; cluster 12 was enriched with genes related to protein localization, some of which may be involved in the transport of proteins into the newly formed synapses; and cluster 6 was related to cell-cell adhesion, a process well known to be involved in both synaptic plasticity [20−21] and synapse formation [22]. The other two clusters in this synapse category – clusters 5 and 9 – were explicitly linked by their annotation terms to synaptic transmission, learning, and memory.

**Table 2:** Annotation clusters of genes up-regulated at Day 5 identified by DAVID.

category	cluster number		enrichment score	number of genes		annotation terms		Gene Ontology ID (or other databases)		p value
synapse		2	4.141		22	neuron projection	GO:0043005		3.32×10^−6^	
					26	cell projection	GO:0042995		1.21×10^−4^	
					11	axon	GO:0030424		9.37×10^−4^	
		4	3.329		43	plasma membrane part	GO:0044459		5.48×10^−6^	
					61	plasma membrane	GO:0005886		2.79×10^−5^	
					49	membrane	PIR keywords		0.676	
		5	3.213		20	transmission of nerve impulsw	GO:0019226		3.58×10^−7^	
					17	synaptic transmission	GO:0007268		1.03×10^−6^	
					20	cell-cell signaling	GO:0007267		3.59×10^−6^	
					10	learning or memory	GO:0007611		4.22×10^−4^	
					13	behavior	GO:0007610		0.0595	
		6	2.594		10	adherens junction	GO:0005912		1.08×10^−4^	
					12	basolateral plasma membrane	GO:0016323		1.33×10^−4^	
					10	anchoring junction	GO:0070161		1.66×10^−4^	
					5	cell-cell adherens junction	GO:0005913		2.41×10^−3^	
					6	cell-substrate junction	GO:0030055		8.59×10^−3^	
		9	2.323		6	regulation of synapse structure and activity	GO:0050803		6.72×10^−5^	
					10	learning or memory	GO:0007611		4.22×10^−4^	
					11	regulation of synaptic transmission	GO:0050804		5.21×10^−4^	
					11	regulation of t*r*ansmission of nerve impulse	GO:0051969		8.61×10^−4^	
					7	learning	GO:0007612		1.40×10^−3^	
		10	2.247		6	regulation of synapse structure and activity	GO:0050803		6.72×10^−5^	
					4	regulation of synaptogenesis	GO:0051963		2.32×10^−3^	
					4	regulation of synapse organization	GO:0050807		3.86×10^−3^	
					4	memory	GO:0007613		0.0730	
					5	regulation of cellular component biogenesis	GO:0044087		0.133	
		12	2.126		16	cellular protein localization	GO:0034613		3.70×10^−4^	
					16	cellular macromolecule localization	GO:0070727		3.96×10^−4^	
					19	intracellular transport	GO:0046907		1.88×10^−3^	
					23	protein localization	GO:0008104		1.90×10^−3^	
					12	intracellular protein transport	GO:0006886		9.96×10^−3^	
development		3	4.021		16	cell morphogenesis involved in differentiation	GO:0000904		1.67×10^−5^	
					19	cell morphogenesis	GO:0000902		2.05×10^−5^	
					22	neuron differentiation	GO:0030182		3.46×10^−5^	
					19	cell projection organization	GO:0030030		4.51×10^−5^	
					16	neuron projection development	GO:0031175		6.75×10^−5^	
		8	2.395		9	eye development	GO:0001654		2.71×10^−3^	
					8	camera-type eye development	GO:0043010		2.87×10^−3^	
					12	sensory organ development	GO:0007423		3.28×10^−3^	
					6	eye morphogenesis	GO:0048592		6.36×10^−3^	
					5	camera-type eye morphogenesis	GO:0048593		6.52×10^−3^	
intracellular signaling		1	4.317		21	protein kinase, ATP binding site	InterPro IPR017441		1.86×10^−8^	
					26	kinase	PIR keywords		2.78×10^−8^	
					21	protein kinase, core	InterPro IPR000719		1.60×10^−7^	
					39	nucleotide-binding	PIR keywords		1.69×10^−7^	
					17	serine/threonine-protein kinase	PIR keywords		2.96×10^−7^	
		7	2.409		18	G-protein modulator	Panther MF00100		2.06×10^−4^	
					15	nucleoside-triphosphatase regulator activity	GO:0060589		2.60×10^−4^	
					13	small GTPase mediated signal transduction	GO:0007264		5.34×10^−4^	
					14	GTPase regulator activity	GO:0030695		6.95×10^−4^	
					11	small GTPase regulator activity	GO:0005083		1.11×10^−3^	
transforming growth factor beta receptor		11	2.200		5	transforming growth factor-beta receptor type I and II	Panther PTHR23255		3.97×10^−5^	
					4	GS domain	Uniprot Sequence Feature		6.94×10^−5^	
					4	TGF beta receptor, GS motif	InterPro IPR003605		8.22×10^−5^	
					4	transforming growth factor beta receptor activity, type I	GO:0005025		9.69×10^−5^	
					5	transforming growth factor beta receptor activity	GO:0005024		1.00×10^−4^	
fibroblast growth factor		13	2.020		10	FGF signaling pathway	Panther P00021		3.47×10^−3^	
					4	fibroblast growth factor receptor signaling pathway	GO:0008543		8.50×10^−3^	
					11	angiogenesis	Panther P00005		0.0296	

The other four categories, all listed in Table 2, were related to development (clusters 3 and 8), intracellular signaling molecules (clusters 1 and 7), transforming growth factor beta receptor activity (cluster 11), and the fibroblast growth factor (FGF) (cluster 13), respectively. Of these 5 categories, the intracellular signaling category was the largest even though it comprised only 2 functional gene clusters. These categories also overlapped with each other considerably (Fig. 8A). For example, most of the genes in the FGF category were also included in the synapse, intracellular signaling, and development categories; the synapse, development, and intracellular signaling categories also shared many common genes as well.

For completeness, we performed an analogous DAVID clustering analysis on the *Sham*-specific genes primarily up-regulated at Day 5 (276 genes). Four significantly enriched clusters were found. Together they comprised 66 genes, or 24% of the modulated probes with gene symbols, a percentage much lower than the proportion of *Reach*-specific genes falling into functional clusters (Fig. 6C and 6E, black bars). Cluster 1 was linked to mRNA processing and RNA splicing; cluster 2 was linked to nucleoplasm, nuclear body, and membrane-enclosed lumen; cluster 3 was associated with anatomical structure homeostasis and telomere organization; cluster 4 contained genes related to mRNA transport and localization. Thus, these functional clusters had completely different annotation terms from those identified from the *Reach*-specific genes, with no term related to the synapse, development, or any growth factor. Some of the *Sham*-specific genes with the most differential expression included *Clk4* (CDC like kinase 4), *Clk1* (CDC like kinase 1), *Iqub* (IQ motif and ubiquitin domain containing), *Dhx15* (DEAH box polypeptide 15) and *Omg* (oligodendrocyte-myelin glycoprotein).

### Synapse Genes were Evenly Distributed Across Probes of Different Fold-Change Values

In our selection of differentially expressed probes described above, we imposed the filtering criterion that the between-group difference had to be greater than 1.5-fold in at least one of the three group-pairs. It is possible that most of genes grouped into functionally-enriched clusters by DAVID were all probes with higher fold-change values, thereby implying that the differentially expressed probes with smaller fold-change values may contain a higher proportion of false positives. We examined the percentage of probes remaining in the synapse-related and other clusters by successively increasing the filtering fold-change threshold from 1.5 to 1.75 and 2.0 fold. As this was increased, the number of unique annotated probes up-regulated at Day 5 decreased from 339 at 1.5 fold, to 143 at 1.75, and down to only 32 at 2.0 fold. However, both the percentage of probes belonging to any functional clusters and the percentage of probes belonging to the synapse category did not change substantially across all three fold-change criteria (Fig. 8B). Therefore, the genes within the enriched clusters were evenly distributed across the probes with different fold-change values, implying that the false positive rate would probably not be much lower even with a more stringent fold-change threshold (assuming that the “true” proportion of "clusterable" genes is itself independent of the fold-change threshold).

A list of selected synapse-related genes up-regulated at Day 5 is provided in Table 3. A full list of all *Reach*-specific differentially expressed genes can be found in Supplementary Materials (Table S1). Note that we only performed functional clustering analysis on the genes modulated at Day 5 but not on those modulated at Day 12 because the number of unique gene symbols present in the latter group was too small (n = 28) for DAVID to yield any statistically meaningful clustering results (see [17], p. 44).

**Table 3:** List of selected genes in the synapse category differentially expressed at Day 5 of motor skill learning.

Gene symbol	Gene name	P-value	Fold Change5-day-Reach w. r. t.		Gene expression intensities (mean±SE)		
			0-day	12-day-Reach	0day	5day-Reach	12day-Reach
tmod2	tropomodulin 2	0.0097	1.30	1.56	0.30±0.11	0.67±0.13	0.02±0.07
ctnnd1	catenin (cadherin associated protein), delta 1	0.0125	1.67	2.06	0.33±0.16	1.07±0.16	0.03±0.14
enah	enabled homolog (Drosophila)	0.0125	1.33	1.78	0.38±0.08	0.79±0.17	−0.04±0.05
rap2b	RAP2B, member of RAS oncogene family	0.0125	1.18	1.61	0.44±0.07	0.67±0.10	−0.01±0.13
cask	calcium/calmodulin-dependent serine protein kinase (MAGUK family), probe 1	0.0132	1.27	1.56	0.35±0.07	0.69±0.11	0.05±0.09
syt1	partial mRNA for synaptotagmin 1 (syt1 gene), splice variant 4	0.0154	1.17	1.57	0.87±0.06	1.10±0.13	0.45±0.09
itga6	Integrin alpha 6 subchain Fragment	0.0173	1.31	1.82	0.16±0.07	0.55±0.13	−0.31±0.16
dmd	dystrophin, transcript variant Dp71c	0.0183	1.44	1.65	0.24±0.06	0.77±0.18	0.05±0.09
epha4	Eph receptor A4	0.0210	1.54	1.64	−0.07±0.08	0.55±0.14	−0.16±0.10
adcy1	adenylate cyclase 1 (brain)	0.0210	1.36	2.52	1.54±0.19	1.98±0.35	0.65±0.15
adnp	activity-dependent neuroprotector homeobox	0.0210	1.18	1.81	0.51±0.20	0.75±0.24	−0.11±0.11
acsl1	acyl-CoA synthetase long-chain family member 1	0.0218	1.23	1.60	0.48±0.09	0.78±0.08	0.11±0.15
dnajc6	DnaJ (Hsp40) homolog, subfamily C, member 6	0.0231	1.28	1.95	0.80±0.11	1.15±0.11	0.19±0.21
prkar2a	protein kinase, cAMP dependent regulatory, type II alpha	0.0231	1.26	2.03	0.65±0.17	0.99±0.25	−0.04±0.09
sptbn4	Sptbn4 protein Fragment	0.0231	1.18	2.16	0.88±0.15	1.12±0.26	0.01±0.10
ephb2	Eph receptor B2	0.0231	1.10	1.88	1.03±0.25	1.17±0.21	0.26±0.17
tjp1	tight junction protein 1, probe 1	0.0264	1.62	1.94	0.03±0.11	0.73±0.23	−0.22±0.16
tgfbr1	transforming growth factor, beta receptor 1	0.0264	1.40	2.11	0.07±0.22	0.56±0.24	−0.52±0.13
strn3	striatin, calmodulin binding protein 3	0.0345	1.44	1.59	0.23±0.15	0.75±0.12	0.08±0.05
shroom2	shroom family member 2	0.0345	1.24	1.63	0.62±0.12	0.94±0.19	0.24±0.07
syngap1	synaptic Ras GTPase activating protein 1 homolog	0.0345	1.13	1.90	1.20±0.15	1.37±0.27	0.45±0.09
stx16	syntaxin 16	0.0366	1.62	2.01	0.30±0.18	1.00±0.23	−0.01±0.14
adam9	ADAM metallopeptidase domain 9 (meltrin gamma)	0.0366	1.39	1.82	0.26±0.15	0.73±0.21	−0.13±0.20
mtpn	myotrophin	0.0366	1.23	1.50	0.08±0.14	0.37±0.16	−0.22±0.07
bcl2l2	Bcl2-like 2	0.0366	1.18	1.69	0.73±0.15	0.97±0.13	0.21±0.13
syt11	synaptotagmin XI	0.0366	1.15	1.55	0.40±0.14	0.60±0.11	−0.03±0.16
tjp1	tight junction protein 1, probe 2	0.0373	1.53	1.59	−0.10±0.13	0.51±0.14	−0.15±0.07
app	amyloid beta (A4) precursor protein	0.0388	1.43	1.88	0.34±0.14	0.85±0.28	−0.06±0.16
akap2	A kinase (PRKA) anchor protein 2	0.0388	1.36	1.97	0.40±0.26	0.85±0.32	−0.13±0.16
nsf	N-ethylmaleimide-sensitive factor	0.0388	1.24	1.74	0.65±0.20	0.96±0.23	0.16±0.20
cask	calcium/calmodulin-dependent serine protein kinase (MAGUK family), probe 2	0.0435	1.48	1.63	0.03±0.21	0.59±0.15	−0.12±0.08
mycbp2	MYC binding protein 2	0.0435	1.41	1.51	0.10±0.13	0.59±0.17	0.00±0.07
acvr1b	activin A receptor, type IB	0.0435	1.21	1.72	0.72±0.13	0.99±0.19	0.21±0.15
kpnb1	karyopherin (importin) beta	0.0488	1.47	2.22	0.65±0.21	1.20±0.24	0.05±0.19
bhlhe41	enhancer-of-split and hairy-related protein 1 (SHARP-1)	0.0488	1.43	2.22	−0.06±0.17	0.46±0.26	−0.69±0.26
mecp2	methyl CpG binding protein 2	0.0488	1.34	1.82	0.48±0.20	0.90±0.23	0.04±0.14
iqgap1	IQ motif containing GTPase activating protein 1	0.0488	1.30	1.54	0.22±0.15	0.61±0.16	−0.02±0.07
lasp1	LIM and SH3 protein 1	0.0498	1.20	1.93	1.12±0.14	1.39±0.26	0.44±0.21
nlgn2	neuroligin 2	0.0498	1.19	1.68	0.80±0.05	1.05±0.23	0.30±0.21
slc12a5	solute carrier family 12 (potassium-chloride transporter), member 5	0.0498	1.19	1.96	0.92±0.21	1.17±0.31	0.20±0.11
arhgef7	Rho guanine nucleotide exchange factor (GEF7), transcript variant 2	0.0498	1.18	1.60	0.40±0.13	0.64±0.11	−0.04±0.15
pvrl1	Nectin-1 Fragment	0.0498	1.13	1.68	0.63±0.17	0.81±0.19	0.06±0.13

### Unclustered Genes as Potential Novel Candidate Genes for Learning

**Figure 9:**
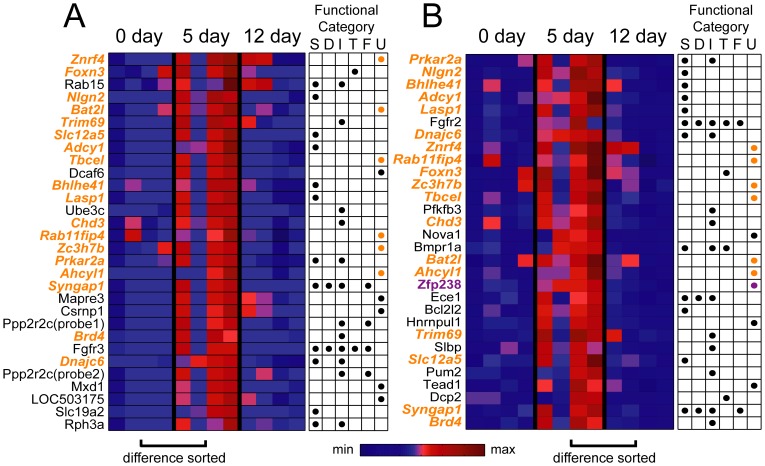
Unclustered genes with the most extreme differential expression as potential new genes related to learning and memory. One way to isolate genes of interest is to select those with the most extreme differences in expression profiles between conditions, assuming that a gene's functional relevance correlates with its magnitude of differential expression. We present here the sample differential expression data of the 30 genes with the most differences in differential expression between 5 days and 0 day (*A*), and between 5 days and 12 days (*B*), in the form of heat maps. Each row of the heat map shows the data of one gene (gene symbol indicated on the left), and the color of each square in the row denotes the expression value of each sample according to a color map with red showing the highest value, and blue, the lowest. The *0-day* columns indicate zero-meaned sample array data (because *0-day* is conceptually the *Sham* of itself) while the 5day and 12day columns indicate the difference between the sample data in the *Reach* group and the mean value of the *Sham* group. To the right of the heat map is a table indicating to which functional categories each gene belonged (S, synapse; D, development; I, intracellular signaling; T, transforming growth factor β receptor activity; F, fibroblast growth factor family; U, unclustered). Gene names shown in orange are the ones common to both lists. The several unclustered genes that show up in both lists are marked with orange dots in the table on the right. In *B*, the gene highlighted in purple (*Zfp238*) is one whose potential roles in learning and memory are further described in Discussion.

Aside from grouping the set of modulated genes into functional clusters, another way to isolate genes of interest is to select those with the most extreme differences in expression profile between conditions, assuming that a gene's functional relevance correlates with its magnitude of differential expression. For genes modulated at 5 days, this means selecting genes with the most differences in differential expression either between Day 5 and Day 0 (i.e., Δ5day; differential expression at Day 0 is by definition zero), or between Day 5 and Day 12 (i.e., Δ5day – Δ12day). Here we show heat maps of the differential expression profiles for the top 30 genes ranked according to both the former (Fig. 9A) and latter (Fig. 9B) criteria, respectively. Eighteen genes were selected by both criteria (Fig. 9, orange gene symbols). Among them, 8 were included in the synapse category (*Nlgn2*, *Slc12a5*, *Adcy1*, *Prkar2a*, *Bhlhe41*, *Lasp1*, *Syngap1*, and *Dnajc6*) while 6 of them did not belong to any of the significantly enriched functional clusters (Fig. 9, orange marks under U, denoting the unclustered category). Interestingly, all of these unclustered genes lack extensive functional characterization. To the best of our knowledge the neuronal functions of Bat2l (proline-rich coiled-coil 2B, or Prrc2b), Zc3h7b (zinc finger CCCH-type containing 7B), and Ahcyl1 (adenosylhomocysteinase-like 1) are yet to be documented. Three of the unclustered genes are potentially related to intracellular protein trafficking: Tbcel (tubulin folding cofactor E-like) acts to depolymerize microtubules and may thus regulate vesicle transport [23]; Znrf4 (zinc and ring finger 4, or Nixin) is an ubiquitin ligase in the endoplasmic reticulum (ER) that may function to prevent an overload of protein influx into the ER by regulating the amount of the chaperon calnexin [24]; Rab11fip4 (Rab11 family interacting protein 4 class II) modulates the activity of Rab11, a small GTPase that in turn regulates vesicle trafficking [25]. One other unclustered gene with potential functional significance is *Zfp238* (zinc finger protein 238) (Fig. 9B, purple gene symbol), which encodes a DNA-binding transcriptional repressor required for the maturation of cortical and hippocampal neurons during development [26] (see Discussion).

### Array Data were Validated by qPCR

**Figure 10:**
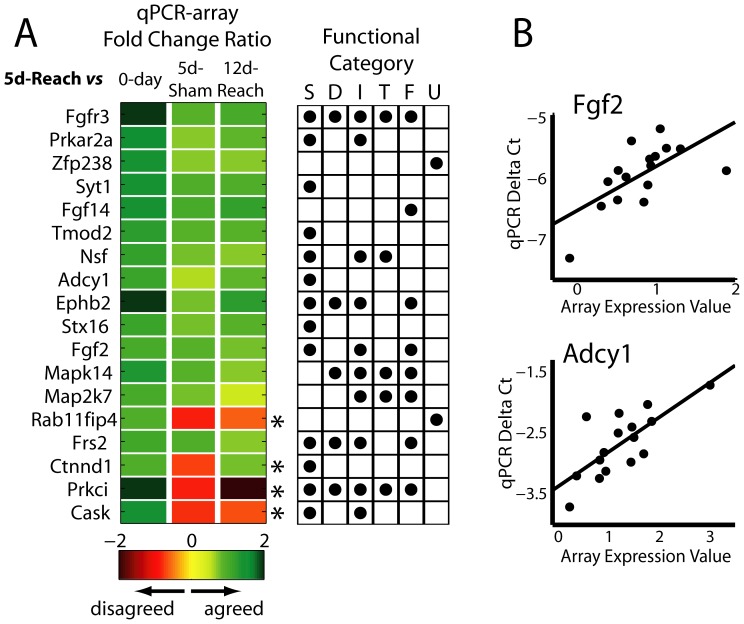
Microarray data were validated using qPCR. *A*, Gene expression fold changes indicated by microarray and qPCR. Eighteen genes (listed on the figure's left) were selected from the synapse, FGF, and unclustered categories for qPCR validation. Array and qPCR fold change values were calculated for each gene for the comparisons, *5-day-Reach* versus *0-day, 5-day-Reach* versus *5-day-Sham*, and *5-day-Reach* versus *12-day-Reach*, resulting into a total of 18×3 = 54 comparisons. The extent of agreement between the direction of fold change values derived from the two methods was assessed by the ratio of qPCR-fold change to the array-fold change. A positive ratio indicates an agreement, and a negative ratio, a disagreement. The heat map on the left shows this ratio of each of the 54 comparisons using a color map with green showing the highest (positive) value, and red, the lowest (negative) value. Four genes (*Prkci*, *Rab11fip4*, *Ctnnd1*, and *Cask*) showed a disagreement in at least one of the three comparisons. These four genes showing mismatches between the array- and qPCR-fold change directions (*) were also the ones whose qPCR-▵C_T_ values of the *5-day-Reach* group were not significantly different from those of the other groups (p>0.05), and whose qPCR-▵C_T_ values did not correlate well with the microarray expression intensities (p>0.05). To the right of the heat map is a table indicating to which functional categories each gene belonged (S, synapse; D, development; I, intracellular signaling; T, transforming growth factor β receptor activity; F, fibroblast growth factor family; U, unclustered). *B*, For some of the genes, we observed an excellent correlation between the sample qPCR ▵C_T_ values and the sample microarray gene expression intensities. They included *Fgf2* (top panel; *r* = 0.64. p<0.01) and *Adcy1* (bottom panel; *r* = 0.73, p<0.01). In both graphs here, the qPCR C_T_ values were normalized with respect to those of the *Ywhaz* gene.

From the list of 339 genes found by our microarray analysis to be up-regulated at Day 5, we selected 18 of them, including 13 genes belonging to the synapse category, 8 genes to the FGF category, and 2 unclustered genes, for further qPCR validation (Fig. 10A, right panel). For each gene, fold-change values comparing expressions of *5-day-Reach* versus *0-day, 5-day-Sham*, and *12-day-Reach*, respectively, were derived from the normalized C_T_ values, resulting in a total of 18×3 = 54 fold-change comparisons. Overall, there was good agreement between the results of these two methods. The qPCR fold-change direction agreed with that indicated by microarray in 47 of the 54 comparisons (Fig. 10A, left panel). Of the 18 genes, 10 of them showed a statistically significant difference (p<0.05) between the normalized C_T_ values of *5-day-Reach* and those of the other conditions. However, among the remaining 8 genes, 2 of them (*Fgf2* and *Mapk14*) had small p values (p<0.08), and 4 of them (*Fgf2*, *Mapk14*, *Map2k7*, and *Frs2*) showed a significant correlation between microarray and qPCR data (p<0.05; p<0.01 for 3 of 4 genes). In fact, the correlation between the microarray gene expression intensities and the qPCR normalized C_T_ values was statistically highly significant for genes such as *Fgf2* (*r = *0.64, p<0.01) and *Adcy1* (*r* = 0.73, p<0.01) (Fig. 10B). Thus, of the 18 genes, only 4 of them – *Rab11fip4*, *Ctnnd1*, *Prkci*, and *Cask* – failed both statistical tests (Fig. 10A, *). The overall good agreement between the microarray and qPCR data supports the quality of our microarray data.

## Discussion

In this study we examined the transcriptome of the forelimb motor cortex at different time points of motor skill learning. We designed a non-trivial forelimb task (Figs. 1, 4) in which the animals displayed a sigmoid learning curve (Fig. 2D), enabling us to profile gene expression before skill training (*0-day*), immediately preceding facilitated task performance (*5-day-Reach*), and after performance reached its peak (*12-day-Reach*). Most of the transcriptional changes occurred at 5 but not at 12 days (Fig. 6C). Our functional clustering analysis further revealed that the set of genes differentially expressed at Day 5 included many that are related to the synapse and the FGF signaling pathway (Fig. 8A; Table 2), as well as several other genes with hitherto un-described roles in memory formation (Fig. 9). Our behavioral and microarray data permit an overview of the molecular and cellular mechanisms driving motor skill learning, and suggest candidate genes which may be key molecular mediators of motor cortical plasticity.

### Genes Modulated by Motor Learning are Involved in Synapse Formation and Plasticity

In our gene expression analysis, many genes up-regulated at Day 5 were categorized by the DAVID algorithm into functional clusters related to the synapse (Fig. 8A, synapse category). Many of these genes (Table 3) have in fact been directly linked to synaptogenesis or dendritic spine formation in previous studies. For example, EphB2 is a receptor tyrosine kinase whose knockdown in cultured cortical neurons reduces the number of dendritic spines, but whose overexpression increases spine density [27]; Iqgap1 is an actin-binding protein whose knockout in the mouse results in a decreased number of spines in both the amygdala and hippocampus [28]. Interestingly, there are several genes in the list that appear to be negative regulators of synapse formation. For example, Syngap1, a major component of the post-synaptic density at glutaminergic synapses, has an expression peak during development that coincides with the time of synaptogenesis [29], but its deletion in the mouse leads to an accelerated formation of dendritic spines that are larger than the normal size [30]. Other genes in the synapse category may also play a role in synapse formation by virtue of their established roles in neurite outgrowth. For instance, Arhgef7 has been shown to be a key signaling molecule during neurite extension induced by the fibroblast growth factor 2 (FGF2) [31]; Enah is a protein implicated in the spatial control of actin assembly [32] whose downregulation leads to axonal retraction [33].

In addition, some of the synaptic genes we have identified interact with the actin cytoskeleton directly, and others function to regulate the stability of actin filaments. These genes may therefore modulate either synapse formation or synaptic plasticity through their possible roles in modifying spine morphology. For instance, Shroom2 is a myosin- and actin-binding protein that protects F-actin from disruption [34]; Lasp1 is known to be a regulator of actin polymerization and cell motility in nonneuronal cells, but also demonstrated to be highly concentrated at cortical synaptic sites [35].

Other genes in the synapse category are related to regulations of synaptic plasticity. Two genes in the list, in particular, are related to LTP induction: Adcy1, a membrane-bound enzyme that catalyzes the formation of cAMP, and Prkar2a, a regulatory subunit (type IIα) of protein kinase A whose activation by cAMP leads to phosphorylation of the glutamate receptor 1 of the AMPA receptor, which in turn results in enhanced synaptic transmission due to an increased incorporation of AMPA receptors into the membrane.

The observation that many modulated genes are related to synaptogenesis and synaptic plasticity suggests that very likely, there is active remodeling of neuronal circuitry in the motor cortex at Day 5 when task goal achievement was about to increase notably.

### Reorganization of Cortical Circuitry Driving Skill Acquisition

As revealed by our clustering analysis and our examination of the functions of individual genes, many of the genes differentially regulated prior to skill improvement are known to be involved in synapse formation, neurite outgrowth, regulation of cytoskeletal dynamics, or neuronal plasticity. Given previous demonstrations of how motor skill acquisition may be underscored by both synaptogenesis [9−10], [36] and strengthening of synaptic connections [7−8] in the motor cortex, the transcriptional activation of the synaptic and plasticity genes we observed likely reflects ongoing modification of motor cortical circuits. The up-regulation of the positive and negative regulators of spine density we have identified, for instance, may contribute to an increased rate of spine formation and pruning, respectively; the genes related to cytoskeletal dynamics and synaptic plasticity may function either to stabilize synapses assembled on the newly formed dendritic spines, or strengthen existing synaptic connections through an LTP-like mechanism (as suggested by the qPCR-validated up-regulation of *Adcy1* and *Prkar2a*, two genes related to cAMP signaling). These neuronal activities then lead ultimately to a reorganization of the circuitry responsible for the acquisition of new motor skills.

Importantly, in our data set, differential expression of genes for circuitry reorganization happened at Day 5, a time when task performance was still at the baseline level, about to improve, but not at Day 12 when performance had reached its peak. Recent imaging studies on the mouse sensorimotor cortex have suggested that circuitry remodeling can be initiated quickly in response to training as new dendritic spines appear within an hour after the first learning session [10], [37]. Our results further suggest that as soon as training commences the motor cortex may enter into a state of continuous remodeling, maintained by changes in the neuronal transcriptome, at least until performance starts to improve, on the condition that the subject continues to practice regularly in between.

We speculate that for any given skill, performance improves only after sufficient modifications in the cortical circuitry are accumulated; the extent and duration of remodeling needed may depend on prior experience, talent, or the level of difficulty of the task. This interpretation thus supports the notion that changes in the motor cortical connective pattern is not just the result of skill learning, but participate actively in driving behavioral changes. Alternatively, this gradual remodeling may reflect any optimization of posture or trajectory, necessary for performance improvement, that happens before the success rate increases (Fig. 2B). Such a gradual modification of M1 circuitry may allow the emergence of a new muscle synergy not normally used in the subject's movement repertoire, but critical for the new skill being acquired.

### Novel Molecular Mediators of Motor Skill Learning?

Another notable finding of our study is that the expressions of a number of genes related to the FGF family were up-regulated in the motor cortex after 5 days of training, as indicated by both our array and qPCR data (Fig. 10). These include genes for two ligands, FGF2 and FGF14, and two receptors, FGFR2 (isoforms a and b), and FGFR3. The FGFs constitute a family of 22 cytokines whose signaling in the developing nervous system controls diverse processes such as neural induction, neural patterning, and axonal guidance [38]; a subset of them, including FGF2 and FGF14, are believed to play a role in learning and memory in the adult brain [39].

Given the roles of the FGFs in neural development, we think that the differentially expressed FGF ligands and receptors we have identified likely contribute to circuitry remodeling in the motor cortex during skill learning. In particular, FGF2 secreted from the postsynaptic target has been shown to increase axon branching, increase the size of growth cones, and promote rapid growth of filopodia in both cortical [40] and hippocampal [41] cells; and externally applied FGF2 can induce clustering of synaptic vesicles and presynaptic localization of voltage-sensitive calcium influx in cultured spinal neurons [42]. Also, the FGFR2 receptor (isoform b) on the cerebellar mossy fibers binds with FGF22 derived from the granule cells to induce signals for presynaptic organization [43]. These previous results on FGF functions and our gene expression data together implicate members of the FGF family to be possibly important molecular mediators of motor skill learning through their roles in the growth and differentiation of axon terminals. Interestingly, a previous microarray study focusing on transcriptional changes in the hippocampus during spatial learning also finds one FGF member (FGF18) to be prominently up-regulated after learning [44].

In addition to genes related to the synapse and the FGF family, there were a number of other up-regulated genes whose functions in the brain have until now not been well-documented. The proteins encoded by these genes may well be candidate molecules whose potential roles in memory formation deserve further examination. One such molecule is the zinc finger protein Zfp238 (also called RP58) whose up-regulation at Day 5 is supported by both our array and qPCR data (Fig. 10A). A DNA-binding transcriptional repressor, Zfp238 is highly expressed in the cerebral cortex in the embryonic brain, and specifically in glutaminergic neurons in the adult brain [45]. In the developing cortex, this protein functions to control cell division of progenitor cells and promotes survival of post-mitotic cortical neurons [26]. It also permits the growth of skeletal muscles by repressing the transcription of two inhibitors of myogenesis [46]. The gene's prominent expression in glutaminergic neurons and its roles in neural and muscular development both suggest that it may also regulate the transcription of other genes related to learning and differentiation in the adult cortex.

### Genes Differentially Expressed in the *Sham* Groups

Even though the *Sham* animals were not exposed to any forelimb skill training, our microarray analysis has identified a sizable number of differentially expressed probes in the *Sham* groups (Fig. 6A). Importantly, when these *Sham*-specific genes were subject to bioinformatic functional clustering, not only were the resulting gene clusters completely different from those identified from the *Reach*-specific genes, they were also not explicitly linked to any neuronal or cognitive processes. This difference in functions between the *Reach*- and *Sham*-clusters suggests that the *Reach*-specific differential expression of the synapse- and growth-factor-related genes are likely not a trivial consequence of executing the task contingency related to trial initiation, or the mere passage of time.

While data from the *Sham* animals control for any potential changes of the transcriptome related to the task contingency and the passing of time, the existence of modulated probes in the *Sham* groups still demands an explanation. Since the pellet retrieval task for the *Sham* animals was trivial, they invariably received all pellet reward in every session. The *Sham* animals consumed more pellets than the *Reach* animals, but, because of the very small weight of each pellet (20 mg) relative to the regular ration of chow (12-18 g), the overall difference in food intake between groups is expected to be small. In fact, there was no difference in both the body weight, and the change of body weight from the first to last sessions, between the *Reach* and *Sham* groups (data not shown; ANOVA, p>0.05). Thus, the difference in the amount of food consumed is unlikely to be responsible for the *Sham*-specific genes.

**Figure 11:**
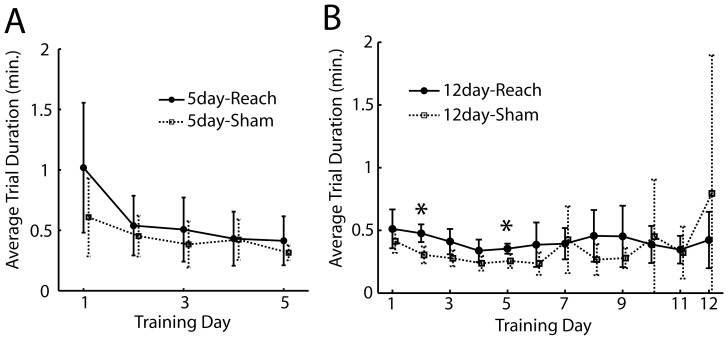
The *Sham* animals might be more motivated to perform the pellet retrieval task then the *Reach* animals. We used the average trial duration as an indicator of the animals' motivation to perform the pellet retrieval task. For both the *5-day* groups (*A*) and *12-day* groups (*B*), trial durations for the *Sham* animals (dotted line; mean±SD; N = 4) tended to be lower than those for the *Reach* animals (solid line; N = 4) even though these differences were not statistically significant for all days except two of the days in the *12-day* groups (*, p<0.05). We speculate that the *Sham* animals might be more motivated or excited to perform the task than the *Reach* animals because successful pellet retrieval was almost guaranteed for the *Sham* groups. This difference in the level of motivation could be an explanation for why we observed many differentially expressed probes specific to the *Sham* groups.

One possibility is that the modulation of the *Sham*-specific genes may be related to the animals' state of arousal. It is possible that the *Sham* animals were more aroused or motivated to perform the task than the *Reach* animals because their reward was guaranteed. If we regard the time needed for a rat to complete a trial as an indicator of its motivation, for both the *5-day* and *12-day* groups, the trial durations for *Sham* animals indeed tended to be lower than those for *Reach* animals (presumably because the *Sham* rats were more motivated to perform the task) even though this difference was statistically significant only for two of the twelve days in the *12-day* groups (p<0.05; Fig. 11, *). Additionally, the known functions of some of the *Sham*-specific genes are consistent with this interpretation. Two of the most differentially expressed *Sham* genes, *Clk4* and *Dhx15*, are more highly expressed in the sparrow brain during wakefulness than sleep [47].

At the very least, the processes driving the modulation of the *Sham*-specific genes likely possess a dynamics very different from that in the *Reach* groups, as suggested by the correlation between Δ5day and Δ12day present only in the *Sham* genes but not in the *Reach* genes (Fig. 6B, 6D). It is also noteworthy that the percentage of probes falling into functional clusters was much lower in the *Sham*-specific genes than in the *Reach*-specific genes (Fig. 6C, 6E). Thus, the whole set of *Sham* genes is less likely to be driven by a unitary input towards a specific biological function.

### Methodological Considerations

Our analysis of the microarray data consisted of two steps. In the first step, probes in both the *Reach* and *Sham* groups differentially expressed relative to *0-day* were isolated. In this identification, we imposed the criterion that the selected probes must have a >1.5 fold change in at least one of the three group-pairs. While making this selection criterion more stringent would certainly reduce the absolute number of false positives, we argue that increasing this threshold would not significantly decrease the rate of false positives (Fig. 8B). In fact, a more stringent selection criterion would decrease the number of differentially expressed genes available for DAVID clustering analysis, thus reducing the power of the enrichment statistics against the genome background [17]. Our selection of the 1.5-fold threshold resulted in ∼300 *Reach*-specific genes, which allowed DAVID to discover 13 significantly enriched clusters that are biologically highly interpretable (Table 2, Fig. 8A).

In the second step of the analysis, differential expression of each *Reach*-specific probe at Day 5 and Day 12 were obtained by subtracting the *Sham* expression values from the *Reach* values. With this procedure, we have essentially defined differential expression at each time point as the gene expression change after skill learning with respect to the expression value without skill learning at the same time point. We think this is a very principled way of obtaining values of differential expression. Our assumption is that all genes modulated by peculiarities specific to the *Sham* groups could be isolated and filtered out as *Sham*-specific genes in the previous analysis step.

When using two-color arrays such as the ones employed here, it is important to account for gene-specific dye biases arising from potentially different amounts of cy3 and cy5 that can be linked to the transcripts of the same gene. To achieve this, we employed a common-reference experimental design so that all 20 cy5-linked samples were hybridized against the same cy3-linked reference isolated from a naïve rat. Any gene-specific dye biases should therefore be present to the same extent in both the *Reach* and *Sham* groups, thus not significantly affecting our isolation of differentially expressed genes. In fact, for Agilent two-color arrays gene-specific dye biases are expected to be very small for all but a few genes [48]. The validity of our profiling results is additionally supported by the consistent data quality across arrays (Fig. 5), the good agreement between the array and qPCR data (Fig. 10), and the observation that the isolated genes could be grouped into biologically interpretable functional clusters.

### A New Rodent Behavioral Paradigm for Studying Skill Acquisition

For this study we introduced a new rodent learning paradigm in which rats were trained to reach and grasp pellets from a randomized, variable location in the workspace. This paradigm thus differs from the standard rodent reaching task in which the animal is trained to reach and grasp from a single, fixed location. We think this design of including a variable reaching target would be useful in other studies as well. This paradigm accommodates inter-animal variation in skill-learning talent by permitting different individuals to excel in different subsets of slots (Fig. 2C). Since different slots also demand differing hand placement strategies for successful retrieval (Fig. 4), it also elicits a wide variety of kinematic patterns, which is important for studies focusing on understanding how the brain and spinal cord control diverse motor behaviors (e.g., [49]). Given the many potential advantages of using rodents in motor control studies [50], our behavioral paradigm could be very useful for future investigations designed for unraveling the neural mechanisms underlying the many behavioral or psychophysical phenomena observed in previous human or non-human primate studies based on multi-directional reaching on a two-link manipulandum (see [51]).

In our behavioral paradigm, our rats showed improvement in task goal achievement following a sigmoid time course (Fig. 2D) rather than the classic power-law or exponential time course. Sigmoid performance has in fact been previously documented in several rat motor learning studies (e.g., see Fig. 3A of [52]; Fig. 3B of [53]; and Fig. 2B of [54]). In a recent modeling paper, Leibowitz *et al.* [19] show that in a task involving successful and failed trials, sigmoid performance is predicted if improvement is driven by the successful trials, but exponential performance results if learning is driven by the failed trials instead. It is thus possible that our rats derive their skill more from the successful reaches, which potentially provide more information for skill acquisition than the failed reaches given that successful pellet retrieval demands definite motor strategies (Fig. 4).

### Limitations and Significance

We have employed the whole-genome microarray technology to search for candidate genes potentially important for motor skill acquisition. The limitations of this approach are well known. The observation that the differential expression of the candidate genes correlated with behavioral changes does not necessarily imply that they have causal roles in driving behaviors. Also, alterations in mRNA levels, as detected by our microarray analysis, do not necessarily reflect similar changes in the amount of their corresponding proteins due to possible translational or post-translational regulations. However, given that we know very little about the molecular biology underpinning motor skill learning, we think it is justified to employ this profiling approach to obtain an overview of the dynamics of transcriptional changes in relation to the time course of motor behaviors, and to screen for promising genes for future experiments. For instance, the expression of a candidate gene we have identified could potentially be manipulated for altering the circuitry in the motor cortex during skill training, so that new insights into the functional roles of the motor cortex during motor skill acquisition may be gained. The candidate genes we have uncovered could also be novel molecules to target for treating motor dysfunction resulting from cortical damage. In fact, there is some behavioral evidence that exogenous application of FGF2, one of the molecules we have identified here, may facilitate motor recovery after cortical injury [55].

*

In summary, our behavioral and gene expression analyses support the idea that continuous circuitry reorganization in M1, maintained by changes of the transcriptome, actively participates in improvement in motor skill performance. Our microarray data further implicate selected members of the FGF family of ligands (FGF2, FGF14) and receptors (FGFR2, FGFR3) as potential molecular mediators of motor learning. How plastic rearrangement of M1 neuronal networks leads to specific changes in muscle activations for the newly acquired skill will be a fruitful area of future investigation.

## Supporting Information

Table S1 **A list of all probes differentially expressed in the** ***Reach*** **groups but not in the** ***Sham*** **groups.** This list of differentially expressed probes is provided as a supplementary file in the xlsx format, readable by Microsoft Excel. The raw gene expression values of the listed probes for all 20 samples can be found in columns H to AA. Gene expression values of each gene were presented as ratios between signal intensities derived from cy5 (labeling samples from the *Reach*- or *Sham-*rats) and those from cy3 (labeling the reference sample from the naïve rat). Name of the file: Table S1.xlsx.
(XLSX)
